# Searching for EGF Fragments Recreating the Outer Sphere of the Growth Factor Involved in Receptor Interactions

**DOI:** 10.3390/ijms25031470

**Published:** 2024-01-25

**Authors:** Katarzyna Czerczak-Kwiatkowska, Marta Kaminska, Justyna Fraczyk, Ireneusz Majsterek, Beata Kolesinska

**Affiliations:** 1 Faculty of Chemistry, Institute of Organic Chemistry, Lodz University of Technology, Zeromskiego 116, 90-924 Lodz, Poland; katarzyna.czerczak@dokt.p.lodz.pl (K.C.-K.); justyna.fraczyk@p.lodz.pl (J.F.); 2 Division of Biophysics, Institute of Materials Science and Engineering, Lodz University of Technology, Stefanowskiego 1/15, 90-924 Lodz, Poland; marta.kaminska@p.lodz.pl; 3 Department of Clinical Chemistry and Biochemistry, Medical University of Lodz, Narutowicza 60, 90-136 Lodz, Poland; ireneusz.majsterek@umed.lodz.pl

**Keywords:** the outer sphere of the protein, polyclonal antibodies, protein fragment microarray, interaction with the receptor, regenerative medicine, SPOT synthesis

## Abstract

The aims of this study were to determine whether it is possible to use peptide microarrays obtained using the SPOT technique (immobilized on cellulose) and specific polyclonal antibodies to select fragments that reconstruct the outer sphere of proteins and to ascertain whether the selected peptide fragments can be useful in the study of their protein–protein and/or peptide–protein interactions. Using this approach, epidermal growth factor (EGF) fragments responsible for the interaction with the EGF receptor were searched. A library of EGF fragments immobilized on cellulose was obtained using triazine condensing reagents. Experiments on the interactions with EGFR confirmed the high affinity of the selected peptide fragments. Biological tests on cells showed the lack of cytotoxicity of the EGF fragments. Selected EGF fragments can be used in various areas of medicine.

## 1. Introduction

Studying protein–protein interactions (PPIs) is key to understanding the biological roles of the various molecules that exist in living organisms, as well as to understanding cell signaling [1,2,3]. Many important human pathologies, including cancer and neurodegenerative diseases, as well as infectious diseases, result from abnormal PPIs [4,5,6,7,8]. The primary and spatial structures of the outer spheres of interacting components are among the most important features responsible for the interactions between proteins. Exposed amino acid residues are spatially oriented in order to fit with the other protein (for example, a receptor), as in a puzzle. Protein–protein or peptide–protein interactions are based on weak interfaces (hydrogen bonds, ionic bonds, and hydrophobic and/or van der Waals forces). In many cases, the interacting proteins recognize each other based on so-called “hot spots” formed by the fragments of proteins, as well as based on the presence of water molecules at the binding site [9,10,11]. The interactions are connected to the structure of the interacting fragments and the free energy of the formed complex [12,13]. Dynamic PPIs are involved in a wide range of activities and processes, such as signaling and folding [14]. Permanent PPIs have a relatively long half-life, whereas transient protein complexes form and break transiently in vivo [15]. Many proteins contain conserved sequences responsible for molecule folding, thus determining their binding with other proteins. Conserved sequences may be characteristic of one group of proteins (for example, the collagen motif) or common for proteins with diverse functions (for example, calcium-binding motifs). These repeats are present in 49% of eukaryotic proteins [16]. These domains are used for predicting the three-dimensional structure of the molecule, which is one of the factors responsible for interprotein interactions [17]. Stable PPIs are usually present in proteins containing numerous repeats, whereas transient PPIs often arise between globular domains and short linear peptide motifs or small structural epitopes.

Protein–protein interactions can be studied in various ways, including through genetic modifications of protein sequences, measurements using sodium dodecyl sulphate–polyacrylamide gel electrophoresis (SDS), SDS combined with Western blotting (WB) [18,19], and others [20,21,22,23,24]. One of the most essential and high-resolution methods of examining the interactions between proteins is X-ray crystallography [25]. Two-dimensional nuclear magnetic resonance is also useful for detecting weak interactions [26]. Another spectroscopic method is circular dichroism (CD). Other physical techniques used for investigating protein–protein interactions include the high-throughput dynamic light scattering method [27,28]. Thermal shift methods, such as differential scanning fluorimetry, differential static light scattering [29], and surface plasmon resonance [30], are valuable techniques for studying protein stability and interactions. Theoretical techniques such as molecular modeling are useful for initial investigations of protein interactions. From theoretical experiments, Kd or second virial coefficient (B_2_) parameters provide information about the characteristics of binding [31]. One of the latest methods for determining protein–protein interactions is microscale thermophoresis (MST) [32]. The development of mass spectrometry has also enabled the study of interacting proteins [33,34,35,36,37]. Various bioinformatic methods are available for the study of protein–protein interactions [38].

While many PPI studies use whole proteins, the use of peptides has increased [39,40,41,42,43,44]. A significant advantage of using peptides over the protein pull-down method is the ability to incorporate post-translational modifications (PTMs). The binding surface can therefore be fully modified [45,46,47,48]. Many proteins are characterized by the presence of specific folded areas determined by the amino acid sequence. Their opposites are regions lacking a specific fold and are referred to as intrinsically disordered regions (IDRs) [49,50,51,52]. Despite their lack of spatial order, IDRs are important elements for the docking of many proteins [53,54]. They often also contain PTMs [55,56,57]. IDRs may contain many interaction motifs, which can be divided into three groups: short linear motifs (SLiMs), molecular recognition features (MoRFs), and intrinsically disordered domains (IDDs) [58,59,60,61,62,63,64,65,66]. Due to the possibility of obtaining modified SLiMs synthetically, they are often used in studies of the impact of post-translational modifications on the interaction capacity of proteins [67,68,69]. Peptides that are SLiMs of the endothelial growth factor receptor (EGFR) containing PTMs have also been studied [70].

Progress in peptide synthesis using the SPOT method has enabled the production of arrays containing many different peptides on a solid membrane [71]. The cellulose membranes used in SPOT synthesis are an attractive alternative to technologies based on polymers, as they can be directly used in biological tests, including immunoassays or pull-down methods [72,73]. SPOT synthesis allows for the incorporation of various PTMs into peptides immobilized on a cellulose membrane, enabling systematic comparisons of modified and unmodified peptide sequence interactions. Cellulose matrices with attached peptides are widely used in PPI research [74,75,76,77,78,79]. The PrISM (Protein Interaction Screen on a Peptide Matrix) technique is based on the use of peptide arrays of overlapping immobilized peptides on solid membranes covering the entire protein sequence. These peptide matrices are a sliding reading window for detecting interactions [40].

Epidermal growth factor (EGF) is a short, 53-amino acid polypeptide with a number of biological actions. It is essential to living organisms because it regulates many functions in cellular processes, including proliferation and differentiation. EGF is present in many mammalian body fluids and tissues [80,81]. For example, its presence in saliva induces the regeneration of gastric mucus and has a protective effect. EGF is also responsible for the regeneration of gastric ulcers [82]. EGF alone or in combination with other drugs may accelerate the regeneration of digestive system wounds [83]. EGF is released by cells and then binds to the EGF receptor (EGFR, ErbB-1) located on the cell surface. EGFR is expressed in a wide range of tissues, including normal and malignant tissues [84], which shows the importance of EGF in tumor-related processes. The excessive expression of EGFR is found in various types of cancer, including lung, kidney, breast, head, and neck cancers [85]. The overexpression of EGFR in a number of tumors promotes the susceptibility of cancer cells to small concentrations of EGF. These cells are able to secrete their own EGF, which, combined with the lower demand for this polypeptide, makes cancer cells proliferate and multiply more quickly. A lower EGF concentration in the brain is associated with neurodegenerative diseases [86]. Thus, low EGF plasma levels may act as a predictive biomarker of long-term cognitive decline in both Parkinson’s disease and Alzheimer’s disease [87]. EGF is widely researched due to its function as a promotor of tissue regeneration. Topical treatment with EGF enhances wound healing. Treatment with EGF also increases collagen synthesis around the wound [88]. EGF is registered as a drug for diabetic foot ulcer treatment. When injected locally into the wound, it accelerates regeneration and helps prevent the need for amputation [89]. The topical treatment of diabetic foot ulcers with EGF has also shown promising results [90]. Given its molecular structure, receptor binding properties, and pivotal role in cell proliferation and differentiation, combined with its medical uses, further research is justified into the biological mechanisms of EGF. In the case of EGF, there are only three binding sites interacting with a total of 16 residues [91]. Ogiso et al. [91] studied the crystal structure of EGF:EGFR, showing the formation of structures containing dimerized EGFR bound with two EGF chains. They identified three binding sites for EGFR and presented the interactions that occur between amino acids during the formation of the EGF:EGFR dimer complex. The properties of the short peptide fragments of proteins and their susceptibility to proteolysis may be modified by incorporating functional groups into its backbone. The aim is to find the shortest protein fragment that can interact with the target protein.

The aim of this study was to find the shortest EGF fragments responsible for EGF–EGFR interactions. We used a peptide library immobilized on a cellulose matrix and employed polyclonal antibodies to determine whether the outer sphere of a protein is responsible for the same interactions with both polyclonal antibodies and the receptor. We expected that an approach using polyclonal antibodies to reconstruct the outer sphere of a protein would be a useful method for studying protein–protein interactions. Moreover, we explored whether its reconstitution preserves their protein-binding affinity. Peptides with such preserved biological activity could have various therapeutic applications. Knowing which part of a protein is responsible for interactions with the receptor will pave the way for designing protein sequences that can either enhance or inhibit its interactions with the receptor, as well as modify its responses to ligands.

## 2. Results and Discussion

**Figure 1:**
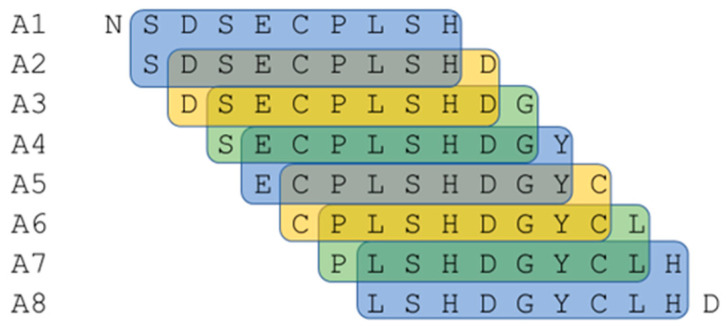
Part of the library of EGF peptides synthesized using SPOT synthesis. Common amino acids in adjacent peptides are color-coded. A1–A8—positions of the peptide on the cellulose matrix. Amino acid sequences in peptides are written in a single-letter code.

In our research, we assumed that the outer sphere and exposed parts of protein/peptide chains are responsible for both binding with the receptor and immunization during the production of polyclonal antibodies. Therefore, we hypothesized that polyclonal antibodies could be used to rapidly identify sequences that are likely to be responsible for protein interactions and binding with the receptor. The optimal tool for the efficient synthesis of peptide libraries is SPOT synthesis. This technique was first described by Frank [92] and later automated. In our research, we used the SPOT method to obtain a library of peptides derived from EGF [93]. A library of peptides constituting the whole sequence of the EGF polypeptide was prepared by dividing the EGF sequence into decapeptides with a frameshift of one amino acid, resulting in overlapping sequences. Each consecutive decapeptide had nine amino acids in common with the previous and next decapeptides, as shown in Figure 1. All synthesized sequences are presented in Table 1.

**Figure 2:**
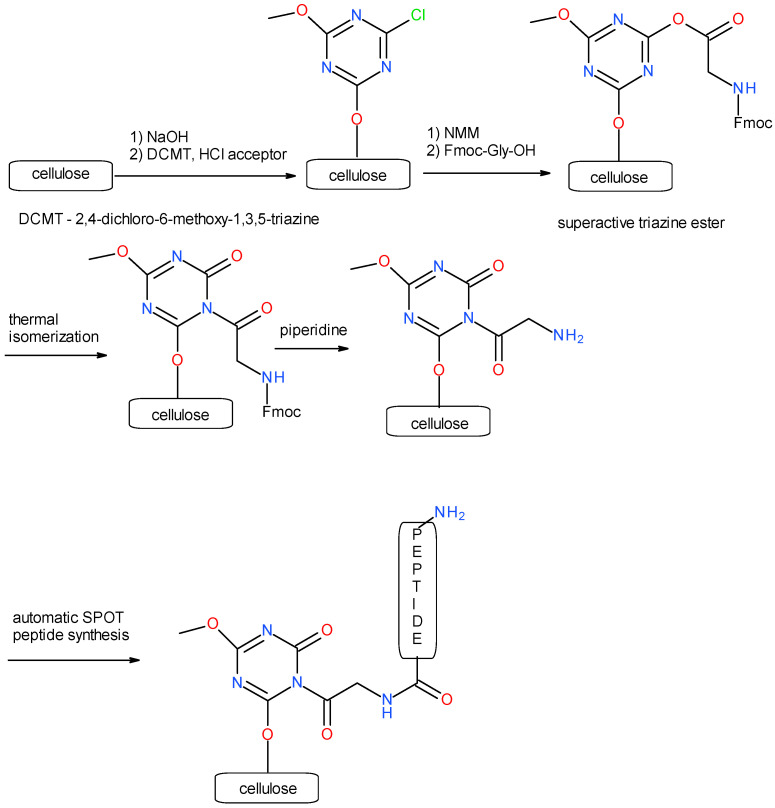
Structure of a triazine linker connecting cellulose and an immobilized peptide.

SPOT synthesis was performed using a ResPep SL automatic synthesizer by Intavis (Tübingen, Germany). The peptides were synthesized on modified cellulose sheets using a triazine linker [93] and DMT/NMM/TosO^−^ as a coupling reagent [94] (Figure 2).

The SPOT synthesis of peptide microarrays is widely used for the rapid synthesis of peptide libraries, which may be later used in various assays, for example, to test interactions between numerous peptides at once, with a diversity of chemical or biological agents. SPOT synthesis has been adapted for different uses. Boisguerin et al. [95] demonstrated methods for synthesizing peptides with free C-termini instead of N-termini, which are more exposed in classic synthesis, allowing for the study of interactions involving this end [95]. Another way to use SPOT synthesis is to design arrays of molecular receptors for the rapid screening of interactions with anti-histamine compounds [96]. Peptide chains synthesized using SPOT synthesis can imitate the binding pockets of receptors. Competitive binding tests can then be used to analyze the biological activity of immobilized peptides [97]. SPOT synthesis has lower reagent usage than synthesis on a larger scale. It is also faster in synthesizing libraries of hundreds or thousands of different peptides, with the added advantage of an easy subsequent blotting analysis. The typical matrix for SPOT synthesis is a modified cellulose sheet, but different solid supports are also used [98]. Prior to synthesis, the cellulose membrane must be modified with a spacer. This spacer links the peptide to the matrix and ensures adequate separation, maintaining an optimal environment for further reactions. Depending on the purpose, different linkers may be used, such as β-Ala-β-Ala [99] or its derivatives. Linkers can also be designed to detach under certain conditions, for example, a change in pH [100]. Fraczyk et al. presented a cleavable linker derived via glycine attachment to an isocyanuric ring, which can be used in the automatic synthesis of peptide arrays [101].

The modification of the cellulose matrix and the automatic synthesis of immobilized EGF fragments are presented in detail in Figures S1 and S2 in the Supporting Information.

**Figure 3:**
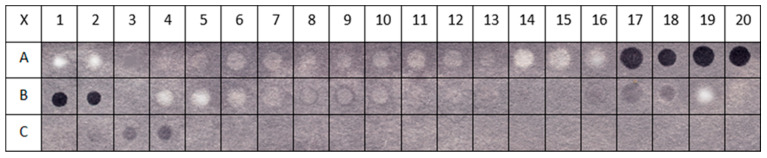
Spot localization on membranes after SPOT synthesis of immobilized peptides and dot blot tests. The numbers 1–20 and letters A, B, and C represent 44 decapeptides of EGF attached to cellulose membranes, probed with a polyclonal antibody to EGF. Each individual spot (dot), which is a single peptide, has its own characteristic index. The peptide sequences are listed in Table 1. For example, the peptide in spot B1 has the amino acid sequence MYIEALDKYA.

Dot blot reactions were performed using primary anti-EGF rabbit polyclonal antibodies (Abcam 9695) (Cambridge, UK) and secondary goat anti-rabbit antibodies conjugated with HRP (Abcam 205718). The procedure for using the dot blot method to analyze peptides immobilized on a cellulose matrix through a triazine linker was optimized in a previous study by the authors [93,101] and ensures the elimination of non-specific impacts. The results of the dot blot test are presented in Figure 3.

Figure 3 shows cellulose membranes modified with 2,4-dichloro-6-methoxy-1,3,5-triazine with EGF peptides attached to it using SPOT synthesis. Each dot equals one decapeptide. The set of all 44 decapeptides forms a map of EGF. During the analysis of the dot blot results, more focus was placed on the exposed parts of the internal protein sequence, especially the decapeptides built from amino acids that form loops in the native protein.

To quantify the color intensity of the complexes formed between the anti-EGF rabbit polyclonal antibodies and EGF fragments, a procedure was used that included scanning the cellulose sheet and transforming the coloring of individual spots into numerical values on a 256 gray scale. The complexes of anti-EGF rabbit polyclonal antibodies with EGF fragments were divided into four groups: very strong (++), for which the staining intensity value was >120; strong (+), for which the staining intensity value was in the range of 120–90; moderately strong (+/−), for which the color intensity value was in the range of 90–45 and characterized by having no ability to influence; and (−), where the color intensity value was <45.

**Figure 4:**
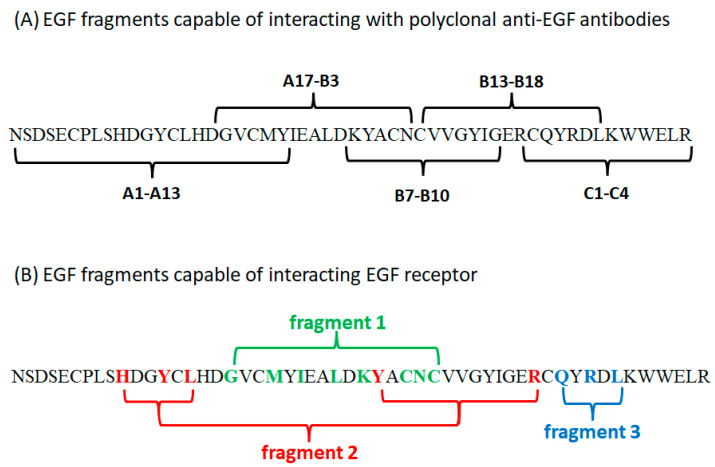
Fragments of EGF responsible for interacting with proteins: (**A**) fragments capable of interacting with specific polyclonal antibodies (fragments found by us), (**B**) fragments involved in binding with EGFR (fragments found by Ogiso et al. [91]). Amino acid residues marked in color are key for interactions.

The results show that there is a region covering A17-B3 decapeptides that is characterized by a high affinity to anti-EGF antibodies. This region stands for the sequence ^17^DGVCMYIEALDKYACN^32^ and is part of the most exposed loop in the native EGF protein. Another active region included peptides B13–B18 with the sequence ^33^CVVGYIGERCQYRDL^47^ and B7–B10 with the sequence ^27^DKYACNCVVGYIG^39^. Other regions showed lower affinity for anti-EGF antibodies or were not active at all. For peptides A1–A13 and C1–C4, the interaction with polyclonal antibodies was weaker but still present. The sequences of the EGF fragments capable of interacting with polyclonal anti-EGF antibodies are presented in Figure 4A.

We next compared the fragments that we found and that interact with polyclonal antibodies from the outer sphere of EGF with the fragments found by Ogiso et al. (Figure 4B) [91]. Ogiso et al. identified three binding sites of EGFR and delineated the interactions between the amino acids that participate in the formation of the EGF:EGFR complex. Their results overlap with those presented in our work. A comparison of the sequences involved in receptor binding with the fragments identified via interactions with polyclonal antibodies is presented in Table 2 and Figure 4B. Considering that the EGF fragments that we found are identical to those described in the literature, it can be assumed that they are able to interact with the proper receptor and induce the same biological effect as the native protein.

The results of our research overlap those reported by Ogiso et al. [91]. The greatest similarity is visible in peptides A17–B3. This fragment is almost identical to fragment 1, capable of interacting with EGFR. This EGF region has the highest affinity to antibodies, especially peptides A20–B2 (color intensity above 120). Peptides A17–A19 are also characterized by a high affinity for polyclonal anti-EGF antibodies (the color intensity ranges from 88.9 to 128). However, peptide B3 shows a lower affinity for antibodies, but the staining is still visible (staining intensity value = 63.8). For peptide A16, a low affinity is found (stain intensity = 44.8). This decapeptide lacks ^26^L, which, according to Ogiso et al. [91], is essential to bind with the receptor. Peptides A15 and B4 show no affinity for antibodies. B4 lacks ^23^I, which is evidence that this residue is important for binding EGF to the receptor. Conversely, decapeptide A15 is composed of ^15^L, which is a residue that participates in the binding of different fragments, and three amino acids that are involved in binding at fragment 1, i.e., ^18^G, ^21^M, and ^23^I. The presence of these residues may be not sufficient when we take into account that, at fragment 1, eight residues are involved in binding, and those not present in the decapeptide might be more important for maintaining activity. An analysis of these sequences shows that the residue sequence most involved in binding is ^22^YIEALDKY^29^, which is present in all strongly interacting decapeptides. Taking into account the peptides with slightly lower interactions, we can suggest that fragment 23–26 is the shortest sequence that maintains at least a low interaction with antibodies in the region. By comparing this analysis with the amino acids presented by Ogiso et al. [91], it is determined that residues 23I and 26L may be the most important for binding in this region. As noticed before, a lack of one of these two amino acids (as in decapeptides A16 and B4) reduces interactions.

The second fragment found by us comprises decapeptides B7–B10 and overlaps with a portion of fragment 1 and fragment 2, capable of interacting with EGFR. The highest affinity is presented by decapeptide B9, which stands for ^29^YACNCVVGYI^38^. This fragment has only sequence ^31^CNC^33^ from the residues that take part in binding with the receptor. Decapeptide B8 additionally contains ^28^K. The activity of this fragment may be associated with its structural similarity to other active regions.

The weakest interaction with antibodies is seen for peptides comprising amino acids from fragment 2. This epitope is hardly visible because of its discontinuity, which means that this binding site requires amino acids from different parts of the peptide chain. The presence of ^10^H, ^13^Y, and ^15^L might be not sufficient to obtain a strong interaction. A lack of ^29^Y and ^41^R may be crucial. The three amino acids ^10^H, ^13^Y, and ^15^L are part of one of the fragments found, but decapeptides comprising amino acids A6–A10 do not show a higher affinity than other decapeptides in this region. Peptides A1–A13, which form this sequence, have a very low affinity for antibodies. Decapeptides can form spatial structures when they are immobilized. However, this becomes especially complicated when trying to find an epitope containing amino acids from different regions of the protein. Only decapeptides in positions A5 and A6 show a slightly more intense dot color (intensity of staining = approximately 60). It might be assumed that the presence of two cysteine residues in these sequences allows for the formation of disulfide bridges, leading these chains to present “loop-like” structures. It is also worth noting that the inactive A14 peptide lacks ^13^Y, suggesting that this residue is crucial for maintaining binding. Similarly, A11 lacks ^10^H, though this difference is less pronounced.

The interaction with fragment 3 involves the least number of residues compared to the other fragments. The most crucial residues are ^43^Q, ^45^R, and ^47^L, which are separated by one amino acid each. These residues are found in two peptides, B13–B18 and C1–C4. Despite containing all the amino acids involved in interactions in fragment 3, namely, ^43^Q, ^45^R, and ^47^L, peptides B19 and B20 do not exhibit the ability to interact with antibodies. This may be because these peptides are anchored to the matrix by their C-terminal residue, potentially preventing this crucial sequence from being exposed. As a result, steric hindrance may prevent interactions with antibodies. Peptides C1 and C2 show moderate binding to antibodies, and residues ^43^Q, ^45^R, and ^47^L are present in their sequences. The last two dots (C3 and C4), which contain terminal amino acids that, according to Carpenter et al., do not take part in EGF binding to the receptor, show interactions with polyclonal anti-EGF antibodies.

One possible reason for the observed activity in tests using anti-EGF polyclonal antibodies may be the use of the whole recombinant protein during the immunization stage (obtaining polyclonal antibodies). Based on the PDB model (2KV4), the C-terminal part of EGF is more exposed and isolated from the rest of the polypeptide chain than the N-terminal domain. During the immunization process, these C-terminal amino acids undoubtedly participate in antigen presentation and antibody formation. However, this fact alone does not confirm their role in binding to the receptor. Another reason why these decapeptides show a high ability to interact with antibodies (as evidenced by the intense coloration of dots) may be the positioning of the amino acids. The presence of the last two C-terminal amino acids, ^52^L and ^53^R, causes a shift in the ^43^Q, ^45^R, and ^47^L residues, which are crucial for EGF binding away from the matrix. The reaction with antibodies is therefore not hindered, as it possibly is for the B19 and B20 peptides due to the presence of the long side chains of ^40^E and ^41^R at the N-terminal part.

By testing the binding of the EGF-derived fragments using specific antibodies, we proved that our proposed approach effectively identified the fragments that constitute the outer sphere of EGF because they were recognized by EGF-specific antibodies. We hypothesize that the fragment of the protein forming the outer sphere should also interact with its specific receptor. This assumption was made because of the characteristics of protein–protein (receptor) interactions, where the affinity between the two molecules depends on their spatial structure (for example, a matching shape of the protein and receptor binding pocket), and interactions between proteins depend on the primary structure of the interacting proteins (amino acid sequence). It is also crucial to expose protein fragments (making an outer sphere) for their mutual interaction.

To verify whether the identified peptides forming the outer sphere of EGF are capable of interacting with EGFR, we conducted interaction experiments with EGFR using the microscale thermophoresis analysis (MST) technique. For this purpose, EGF fragments, peptides with locants of 10–22, 13–19, 22–28, 23–26, 34–47, 38–43, 26–34, 10–15, 43–47, and 10–15, with the addition of ^29^Y and ^41^R at the C- terminal side (10–15a), were synthesized following the SPPS procedure, with DMT/NMM/TosO^−^ as a coupling reagent [100]. The purity of the crude products (directly cleaved from the resin, without purification on preparative HPLC) varied from 75 to 97% (Table 3). The LC-MS spectra of the synthesized EGF fragments are presented in Supporting Material (Figures S3–S12).

In the initial stage of our research using the MST method, we first verified the method by examining EGF binding to its receptor. It is known from the literature data that the dissociation constant of this binding interaction varies from 0.2 to 8 nM [102]. According to the literature data, it was assumed that the concentration of the tested EGF fragments should be significantly higher than the concentration of the receptor protein [103]. It is recommended that the concentration of the ligand should be at least 20× higher than the expected dissociation constant. In the experiments, the final labeled EGFR concentration was 25 nM. Due to the fact that the MST tests started with the 23–26 EGF fragment, and, in this case, the Kd constant was determined only at a peptide concentration of 10 mM, a wide range of concentrations (from μM to mM) was also tested for the remaining EGF fragments.

**Figure 5:**
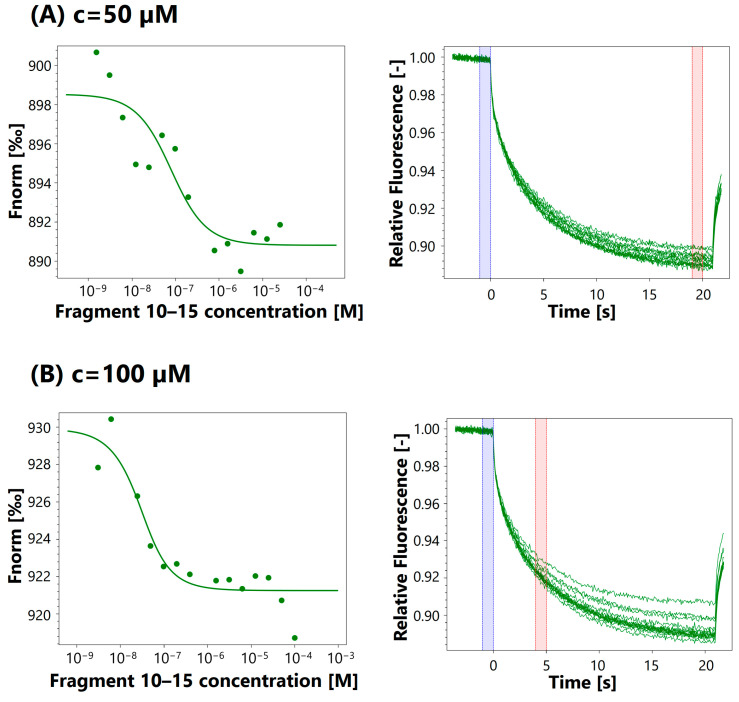

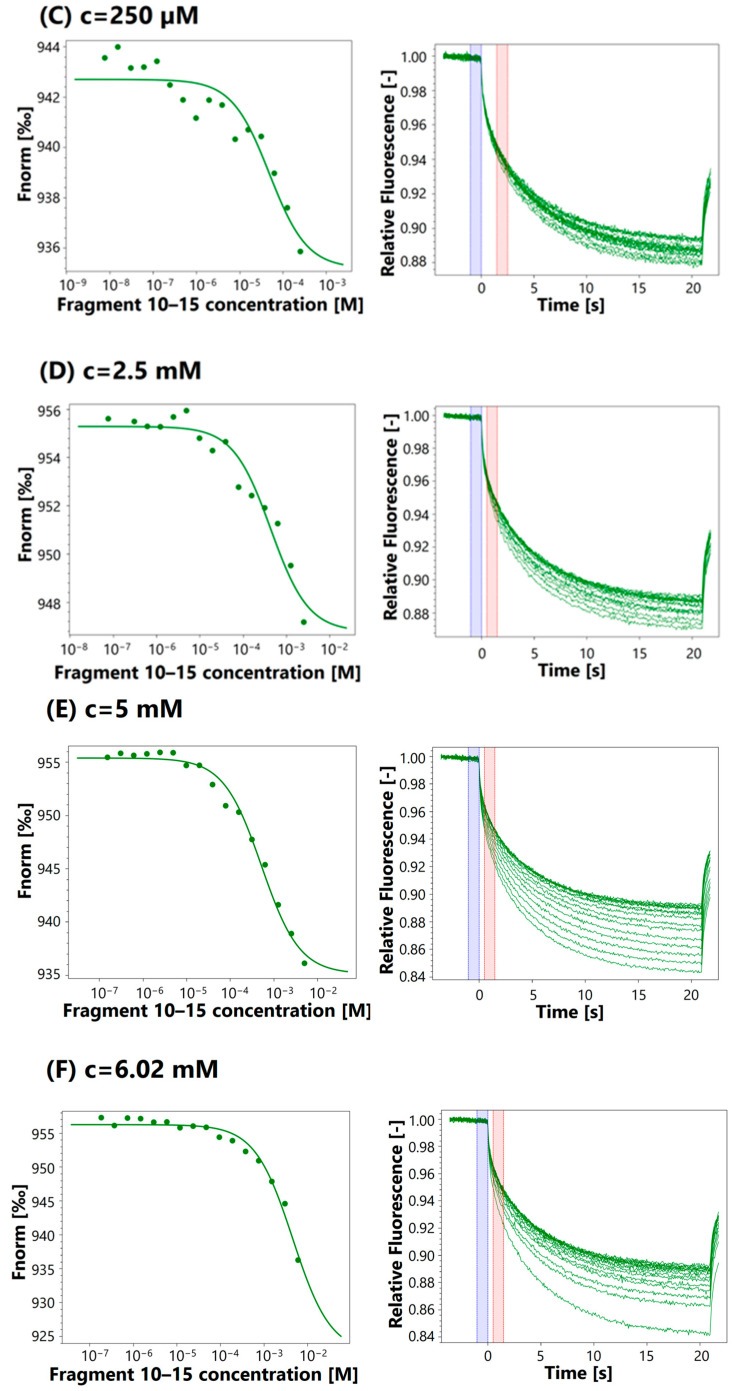

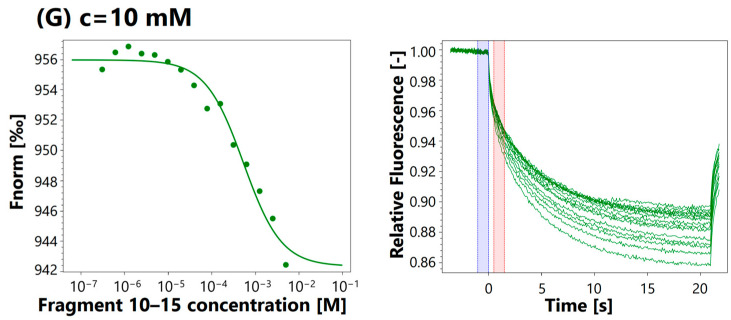
MST analysis for 10–15 EGF fragment (^10^HDGYCL^15^) at different concentrations (c). Assay concentrations (10–15 EGF fragment): (**A**) c = 50 μM, (**B**) c = 100 μM, (**C**) c = 250 μM, (**D**) c = 2.5 mM, (**E**) c = 5 mM, (**F**) c = 6.02 mM, (**G**) c = 10 mM.

The test yielded two comparable dissociation constants: 5.14 nM and 15.8 nM. Given that the Kd values were of a similar order of magnitude, we proceeded with the peptide binding tests. Figure 5 presents exemplary MST curves for the 10–15 EGF fragment (^10^HDGYCL^15^) at various concentrations. MST curves for the other EGF fragments can be found in the Supporting Material (Figures S13–S19).

Fragments 10–22 and 10–15 show binding with dissociation constants of 0.07–416 μM and 48.4–715 μM, respectively, with increasing concentrations (Table 4). The Kd for fragment 10–22 is around 1 order of magnitude lower than the Kd for fragment 10–15. It can be assumed that the higher affinity of fragment 10–22 is caused by the length of the peptide and that this fragment probably resembles native EGF more than the shorter fragment. The second possible reason for the stronger binding of the longer peptide may be the fact that the two last amino acids of this sequence take part in binding to another site, so the formation of the ligand–EGFR complex might be enhanced by binding at two different binding sites at the same time.

Differences in the dissociation constant associated with the peptide length may also be observed for fragments 22–28 and 23–26. Peptide 22–28 shows binding, although with a relatively high Kd of 1.65–1.96 mM and one measurement of 4.02 nM. These values show that the binding is weak. For fragment 23–26, no binding is observed. A dissociation constant of 5.09 mM is obtained only for one measurement, which is higher than that of the longer peptide. This fragment is probably too short to maintain binding. It consists of only two residues that are responsible for binding with the receptor, and it is also probable that the spatial structure cannot be restored.

The binding results for sequences 34–47 (0.5–5.92 μM) and 38–43 (108–709 nM) are of a similar order of magnitude, while sequence 43–47 (4.93–125 μM) binds with higher Kd values. Both 38–43 and 43–47 are part of 34–47. Peptide 43–47 contains the complete sequence responsible for binding at one site, yet it does not bind as efficiently as 34–47 or 38–43. The stronger binding ability of 34–47 is probably connected to its imitation of the three-dimensional structure of native EGF, similarly to the previously described peptides. Sequence 38–43 shows the highest affinity for EGFR, with Kd values of 0.1–1.8 μM. This sequence is part of the fragment selected using the dot blot technique, but it contains only two residues that take part in binding with EGFR, each from a different binding site: 41R and 43Q. Based on PDB (2KV4), this sequence creates a small loop in native EGF. It is possible that the binding of this peptide to the receptor is caused by a combination of its three-dimensional structure and the presence of one of the aforementioned amino acid residues. This could pave the way for the design of novel peptides with sequences that do not only contain conserved amino acids but also only one or two residues that are responsible for binding, combined with the desired three-dimensional structure.

**Figure 6:**
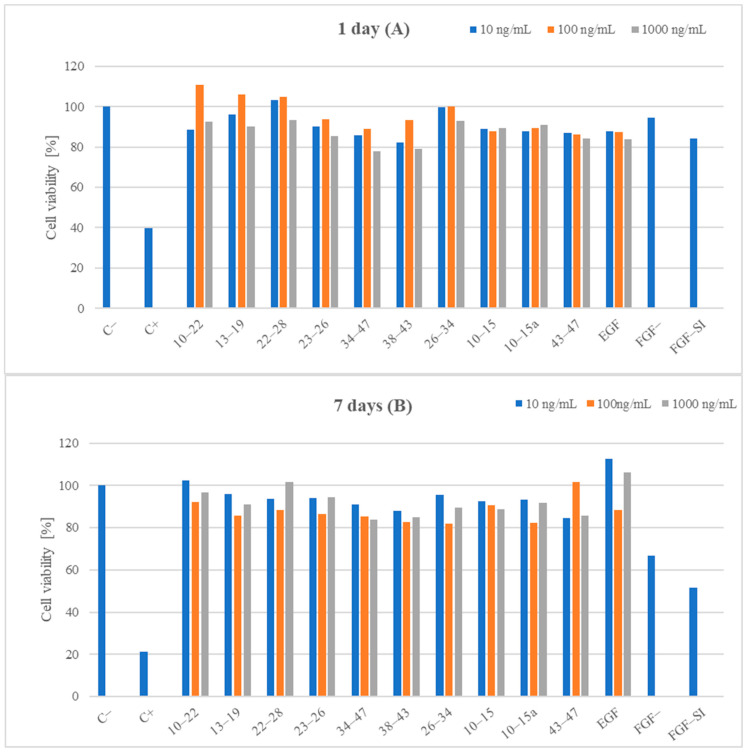
Analysis of the cytotoxicity (at different concentrations) of EGF fragments on Primary Dermal Fibroblasts: (**A**) incubation with EGF fragments for 1 day; (**B**) incubation with EGF fragments for 7 days. EGF-derived fragments were tested at three concentrations: 10 ng/mL, 100 ng/mL, and 1000 ng/mL. C−—negative control, C+—positive control, EGF—native EGF (ab9697). In the cases of FGF– and FGF-SI, cell growth was induced in medium without growth factors (FGF and EGF); in the case of FGF, the added serum may have contained growth factors; in the case of FGF-SI, the added serum was thermically inactivated to inactivate growth factors presented in the serum. The in vitro toxicology resazurin assay was used to assess the cytotoxicity of the tested compounds. All of the experiments were performed in triplicate.

In the next stage of our research, we assessed the safety of selected EGF fragments for medical applications. Although the fragments originated from a native human protein, such an evaluation was necessary because many peptides are known to have cytotoxic activity. Therefore, in the initial step, we investigated the cytotoxicity of all the synthesized fragments (Table 3) using Primary Dermal Fibroblasts (ATCC, Manassas, VA, USA). To evaluate cytotoxicity, we used a resazurin-based assay kit (the resazurin-based assay is a type of colorimetric test). The amount of red intermediate pigment (resorufin) that forms as a result of resazurin reduction is directly proportional to the number of viable cells. The cytotoxicity of the selected EGF fragments was tested at concentrations of 10, 100, and 1000 ng/mL. Cells cultured in a complete growth medium were used as a negative control. Cells incubated with DMSO were used as a positive control. DMSO at a concentration of 1.5% was used in the experiments. According to the literature data [104], low DMSO concentrations (0.01–0.001%) enhance the proliferation of cells when cultured in-vitro, whereas higher concentrations (0.5–3%) lead to a reduction in cell viability in a dose-dependent manner. Finally, no cell survived beyond 3% DMSO. All of the analyzed samples and controls were incubated for 1 day and 7 days (Figure 6).

Figure 6A shows that an incubation period of 1 day at a concentration of 100 ng/mL had the most positive effect on cell viability in the cases of EGF fragments 10–22, 13–19, 22–28, and 26–34, as well as for fragments 23–26, 34–47, and 38–43. For native EGF, the cell viability score was similar at all concentration levels. A similar relationship was observed for fragment 22–28 at a concentration of 10 ng/mL. Fragment 26–34 at concentrations of 10 and 100 ng/mL exhibited cell viability affected in a comparable way to the control sample. Scores around 100% or higher were observed for fragments 22–28 and 26–34 at a concentration of 10 ng/mL and for EGF fragments 10–22, 13–19, 22–28, and 26–34 at 100 ng/mL. However, when all EGF fragments were used at a concentration of 1000 ng/mL, no result higher than 93.5% for fragment 22–28 was observed for any of the peptides. A slightly lower but comparable score was observed for fragments 10–22 and 26–34. At a concentration of 100 ng/mL, only fragment 43–47 had a slightly lower proliferation score than EGF. The other peptides showed similar or higher values. At a concentration of 1000 ng/mL, only fragments 34–47 and 38–43 were characterized by lower proliferation scores than EGF. At a peptide concentration of 10 ng/mL, the same relationship was observed, and only fragments 10–15a and 43–47 had a lower ability to influence proliferation.

Extending the incubation time to 7 days resulted in the highest proliferation values in the peptide trials for 10–22, 13–19, 34–47, 26–34, 10–15, and 10–15a (concentration of 10 ng/mL), as well as for EGF (Figure 6B). A concentration of about 100 ng/mL resulted in the highest cell viability for fragment 43–47. In contrast, cell viability at this concentration was the lowest for fragments 10–22, 13–19, 22–28, 23–26, 38–43, 26–34, and 10–15a, as well as for EGF. For fragments 22–28 and 23–26, the highest proliferation scores were obtained at a concentration of 1000 ng/mL. At concentrations of 10 and 1000 ng/mL, none of the tested peptides showed higher proliferation than EGF. However, at 100 ng/mL, fragments 10–22, 13–19, 10–15, and 43–47 showed higher scores than EGF, and the proliferation score for fragment 22–28 was equal to that for EGF.

None of the tested EGF fragments showed cytotoxic properties at any of the tested concentrations.

**Figure 7:**
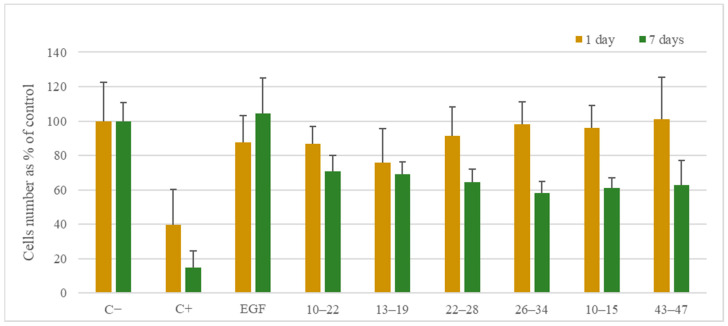
Fibroblast proliferation in culture media after 1 and 7 days in the presence of EGF fragments at a concentration of 100 ng/mL. C−—negative control, C+—positive control, EGF—native EGF (ab9697). Cells cultured in a complete growth medium were used as a negative control. Cells incubated with 1.5% DMSO were used as a positive control.

**Figure 8:**
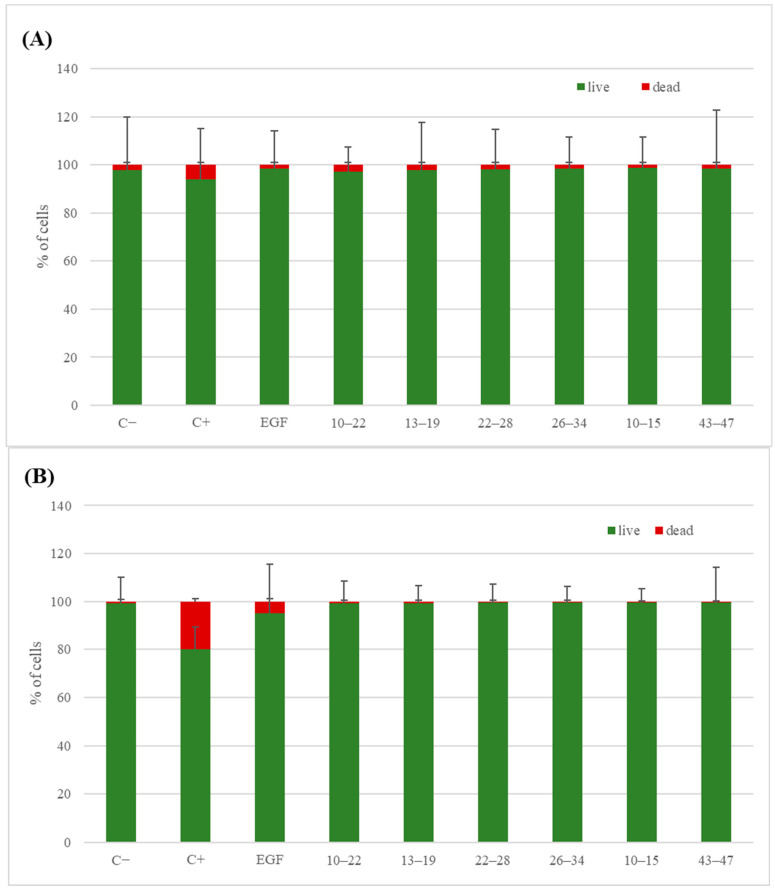
Live/dead assay results after 1 day (**A**) and 7 days (**B**) in the presence of EGF-derived peptides at a concentration of 100 ng/mL. C−—negative control, C+—positive control, EGF—native EGF (ab9697). Cells cultured in a complete growth medium were used as a negative control. Cells incubated with 1.5% DMSO were used as a positive control.

**Figure 9:**
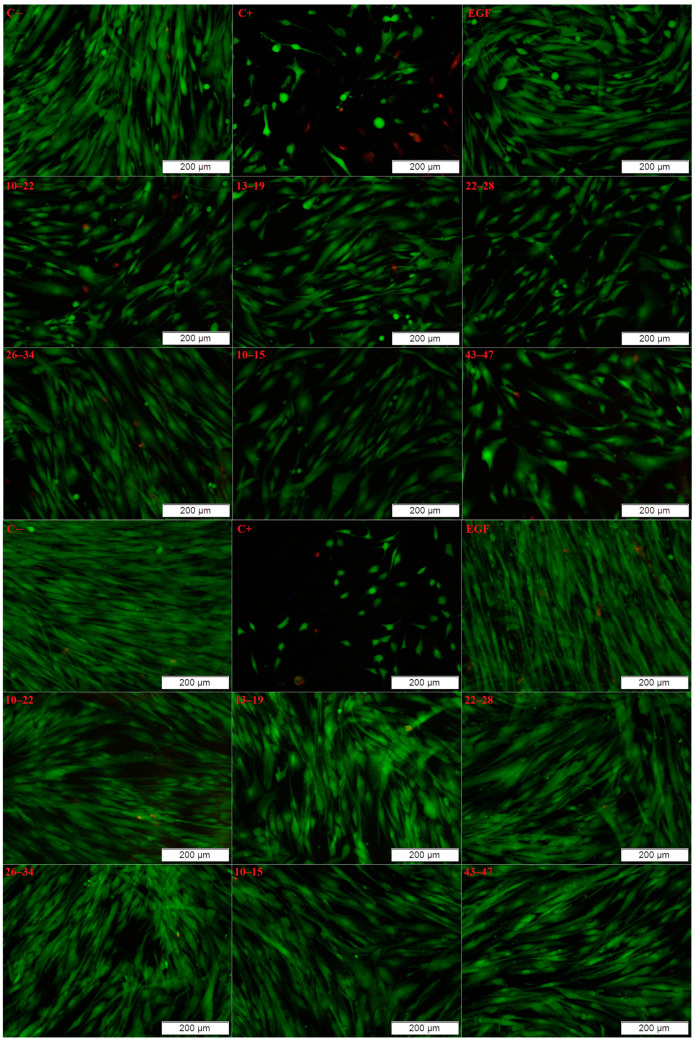
Microscope images of Primary Dermal Fibroblast cells after 1 day (**top**) and 7 days (**bottom**) of incubation with tested EGF fragments. Green corresponds to live cells, and red indicates the presence of dead cells. Cells cultured in a complete growth medium were used as a negative control. Cells incubated with 1.5% DMSO were used as a positive control.

In the next stage of our research, the scope of biological tests was expanded to assess the safety of the found EGF fragments. Fragments 10–22, 13–19, 22–28, 26–34, 10–15, and 10–15a were selected for further study in a live/dead assay (Figure 7, Figure 8 and Figure 9).

For all EGF fragments, the number of cells was lower after 7 days of incubation than in the case of native EGF, which induced cell growth. However, after 1 day of incubation with peptides 26–34, 10–15, and 43–47, there were more cells than in the medium with EGF. The live/dead assay showed that, after 1 day, the percentages of live cells were similar for all EGF fragments added to the cell cultures. In comparison to native EGF, slightly less survivability was observed in the case of supplementation using fragments 10–22, 13–19, and 22–28. After 7 days, the percentages of live cells were all higher with EGF fragments than with native EGF.

The microscope images of the Primary Dermal Fibroblasts (Figure 9) cultured in the presence of EGF fragments indicate their lack of adverse effects on cells. In all cases, the presence of dead cells was marginal. Additionally, incubation in the presence of EGF and EGF fragments did not negatively affect cell morphology.

The results of the viability tests for fragments 10–22, 13–19, 10–15, and 10–15a located in the N-terminal EGF domain show that, after 1 day, fragment 10–22 induced cell growth more than fragments 10–15 and 10–15a, with similar results for fragment 13–19 (concentration = 100 ng/mL). For fragment 10–22 after 7 days, this difference was substantial at a concentration of 10 ng/mL. However, it was less visible for the other peptides. Fragments 10–22, 13–19, and 10–15 were selected for a live/dead assay. Fragment 10–15 presented slightly less cytotoxicity than fragments 10–22 and 13–19 (98.73%, 97.13%, and 97.84% of live cells after one day and 99.52%, 99.25%, and 99.30% after 7 days, respectively). An MST analysis was performed only for fragments 10–22 and 10–15. The results showed that the longer sequence binds more strongly to EGFR than the shorter sequence, by 10 orders of magnitude. The slight differences between the percentages of live cells and the higher binding strength of fragment 10–22 suggest that it could be a potential candidate for therapeutic applications.

From the EGF central fragment pool (fragments 22–28, 23–26, and 26–34), fragment 26–34 in the live/dead test showed one of the highest percentages of live cells after 1 and 7 days. However, an MST analysis was not performed for this fragment. Fragments 22–28 and 23–26 did not show promising results in the MST binding assay. In the proliferation tests, fragment 22–28 resulted in a better yield than fragment 23–26, and the live/dead test for fragment 22–28 showed that the percentage of live cells was favorable compared to the scores for the other peptides.

For the fragments of the C-terminal EGF domain (fragments 34–47 and 38–43), after 1 day of incubation, the proliferation ratio was the highest for 38–43 at 100 ng/mL. However, after 7 days, peptide 43–47 had a better impact on cells than the other two peptides. In the live/dead tests, only 43–47 was selected since this fragment had the highest proliferation ratio at 100 ng/mL after 7 days. This was confirmed by the highest live percentage of cells for all tested peptides after 7 days of incubation at this concentration. During the MST analysis, peptide 38–43 had the lowest Kd of the three peptides, but for peptides 34–47 and 43–47, the results were comparable to those for 10–22, probably due to its length or possession of amino acids essential for binding with the receptor.

## 3. Materials and Methods

LC-MS analysis of peptides was performed using an UltiMate 3000 liquid chromatograph with gradient elution in reversed-phase mode. Phase A—H_2_O LC-MS grade with 0.1% HCOOH; phase B—CH_3_CN LC-MS grade with 0.1% HCOOH. Gradient 10 → 100%B in A (0–3 min 10%B, 3–15 min 10 → 50%B, 15–17 min 50%B, 17–20 min 5 → 100%B, 20–23 min 100%B, 23–27 min 100 → 10%B, 27–30 min 10%B). Column: Kinetex (Phenomenex, Torrance, CA, USA) 2.6u, C18, pore diameter of 100 Å, dimensions of 100 × 4.6 mm. MS was performed with a Bruker micrOTOF-Q III (Bremen, Germany).

A library of EGF fragments immobilized on a cellulose matrix was obtained using an Intavis (Tübingen, Germany) SPOT ResPep SL automated synthesizer.

MST analysis was performed using a Monolith NT.115 device, excitation color—Nano-RED, excitation power—100%, medium MST power.

### 3.1. Synthesis of a Library of Overlapping EGF Fragments Immobilized on a Cellulose Matrix

#### 3.1.1. Immobilization of 2,4-Dichloro-6-methoxy-1,3,5-triazine on Cellulose

Cellulose sheets (Whatman CHR1 filter paper, 2 sheets, 11 × 16 cm) (Little Chalfont, Buckinghamshire, UK) were placed into 1.5 M solution of NaOH (40 mL) for 30 min. The solution was then removed, and the sheets were treated for 3 h with 1 M solution of 2,4-dichloro-6-methoxy-1,3,5-triazine (42.96 g, 0.1 mol) in THF (100 mL) with the addition of NaHCO_3_ (4.2 g, 0.05 mol) and diisopropylethylamine (13.5 mL, 0.075 mmol) for 3 h. The solution was removed, and the sheets were washed with THF (3 × 100 mL, 5 min), THF (100 mL, 30 min), and CH_2_Cl_2_ (100 mL, 2 × 15 min).

#### 3.1.2. Incorporation of Fmoc-Gly-OH on Cellulose as a Fragment of Triazine Linker

Cellulose membranes with immobilized DCMT were treated with 50% *v*/*v* solution of THF:NMM (100 mL) for 30 min. The sheets were then washed with THF (3 × 50 mL) and placed into Fmoc-Gly-OH (8.92 g, 30 mM) and NMM (1.65 mL, 15 mM) in CH_2_Cl_2_:THF (2:1 *v*/*v*, 120 mL) for 1 h. The membranes were washed with CH_2_Cl_2_ (3 × 50 mL) and soaked to remove the excess solvent. The sheets were placed in dry boiling toluene for 8 h and then soaked and dried thoroughly in a vacuum desiccator. The Fmoc group was removed by treatment with a 25% solution of piperidine in DMF (50 mL, 3 × 10 min) and then washed with DMF (3 × 50 mL) and CH2Cl2 (3 × 50 mL).

#### 3.1.3. Synthesis of Decapeptides Using Modified SPOT Technique

For SPOT synthesis, a ResPep SL automatic peptide synthesizer (Intavis, Tübingen, Germany).) was used. For synthesis, 0.56 M solutions in NMP of Fmoc-Ala-OH, Fmoc-Arg(Pbf)-OH, Fmoc-Asn(Trt)-OH, Fmoc-Asp(OtBu)-OH, Fmoc-Cys(Trt)-OH, Fmoc-Gln(Trt)-OH, Fmoc-Glu(OtBu)-OH, Fmoc-Gly-OH, Fmoc-His(Trt)-OH, Fmoc-Ile-OH, Fmoc-Leu-OH, Fmoc-Lys(Boc)-OH, Fmoc-Met-OH, Fmoc-Pro-OH, Fmoc-Ser (tBu)-OH, Fmoc-Thr(tBu)-OH, Fmoc-Trp(Boc)-OH, Fmoc-Tyr(tBu)-OH, and Fmoc-Val-OH were prepared. A 0.5 M solution of 4-(4,6-dimethoxy-1,3,5-triazin-2-yl)-4-methylmorpholinium 4-toluenesulfonate in DMF was used as a coupling reagent, and 2 M NMM in DMF was used as a base. To remove Fmoc protecting groups, a 25% (*v*/*v*) solution of piperidine in DMF was used. To wash the membrane, DMF and dry EtOH were used.

Before the first cycle, the needle was washed inside (1000 μL) and outside (1500 μL), and the membrane was washed with EtOH (2 × 1500 μL).

The first cycle included the following:

Preactivation (6 min, 0.15 μL activator, 0.073 μL base, 0.003 μL NMP, 0.17 μL amino acid); 2 × coupling (15 min); needle rinsing (inside with 500 μL and outside with 1500 μL); extraction (60 s); membrane washing (2 × DMF; 2 × EtOH, per one row 1500 μL); extraction (300 s); preactivation (6 min, 0.15 μL activator, 0.073 μL base, 0.003 μL NMP, 0.17 μL amino acid); 2 × coupling (2 × 3 min); needle rinsing (inside with 500 μL and outside with 1500 μL); membrane washing (4 × DMF; 2 × EtOH, per one row 1500 μL); extraction (60 s); needle rinsing (inside with 500 μL and outside with 1500 μL).

Cycles 2–10 included the following:

Deprotection (2 × 10 min, dispense volume: 500 μL); needle rinsing (inside with 500 μL and outside with 1500 μL); membrane washing (6 × DMF; 2 × EtOH, per one row 1500 μL); extraction (900 s); preactivation (6 min, 0.15 μL activator, 0.073 μL base, 0.003 μL NMP, 0.17 μL amino acid); 2 × coupling (first 3 min, second 5 min); needle rinsing (inside with 500 μL and outside with 1500 μL); extraction (60 s); membrane washing (2 × DMF; 2 × EtOH, per one row 1500 μL); extraction (300 s); preactivation (6 min, 0.15 μL activator, 0.073 μL base, 0.003 μL NMP, 0.17 μL amino acid); 2 × coupling (2 × 5 min); needle rinsing (inside with 500 μL and outside with 1500 μL); membrane washing (4 × DMF; 2 × EtOH, per one row 1500 μL); extraction (180 s); needle rinsing (inside with 500 μL and outside with 1500 μL).

Reports from the automatic SPOT synthesis are included in Supporting Information (Figure S2).

#### 3.1.4. Cleavage of Protecting Groups

Final cleavage of protecting groups was performed manually. The Fmoc group was removed using 25% (*v*/*v*) piperidine in DMF (50 mL, 3 × 10 min), then washed with DMF (3 × 50 mL) and CH_2_Cl_2_ (3 × 50 mL), and dried. The acid-labile protecting groups were removed using a 50% (*v*/*v*) mixture of TFA in CH_2_Cl_2_ (60 mL) with 1% trisopropylsilane (TIS), 2.5% etandithiol (EDT), and 2.5% water for 4 h. For final washing, steps CH_2_Cl_2_ (3 × 50 mL) and EtOH (3 × 50 mL) were used.

### 3.2. Dot Blot Test

Cellulose sheets with attached EGF fragments were treated with PBS buffer (2 × 40 mL, 15 min, pH 7.4). The solution was then removed, and the membranes were treated overnight at room temperature with blocking buffer (3% BSA in PBS, 40 mL, pH 7.4). The cellulose sheets were then washed with PBS buffer (40 mL, 5 min, pH 7.4). Rabbit polyclonal antibodies against EGF (Abcam 9695, 20 μL) were diluted 1:1000 in 1% BSA in PBS (20 mL, pH 7.4) and then incubated with cellulose sheets for 1 h at room temperature. The membranes were then washed with Tween-80 solution in PBS (0.4%, 40 mL, 2 × 5 min) and PBS (40 mL, 5 min). Goat Anti-Rabbit IgG antibodies were conjugated with HRP (Abcam 205718, 20 μL) diluted 1:1000 in 1% BSA in PBS (20 mL, pH 7.4) and then incubated with cellulose sheets for 30 min at room temperature. The membranes were then washed with Tween-80 solution in PBS (0.4%, 40 mL, 2 × 5 min) and PBS (40 mL, 5 min). Visualization of the reactions was performed using 4-chloro-1-naphtol (Sigma–Aldrich). The cellulose sheets were placed into 0.15 mg/mL solution of 4-chloro-1-naphtol in MeOH:PBS (1:5, 15 mL) with the addition of 7.5 μL of H_2_O_2_; reaction time was 30 min. The membranes were washed with distilled water, air-dried, and scanned. In places where immunological complexes developed, blue precipitate was observed. The applied dot blot test procedure ensures the elimination of non-specific interactions. Additionally, using the same SPOT synthesis procedure, a library of type IV collagen fragments was obtained. The use of antibodies against EGF (Abcam 9695) confirmed the lack of binding of the antibodies to fragments of the unsuitable (non-specific) protein, which is an additional factor confirming the correctness of the method used. The gray levels of the immunological reactions were determined using ImageJ Version 1.53n7 (https://imagej.nih.gov/ij/index.html, accessed on 17 October 2021) computer imaging software.

### 3.3. SPPS Synthesis of Selected EGF Fragments

#### 3.3.1. Loading of First Amino Acid

Chloro-(2′-chloro)trityl resin (500 mg) was swollen in CH_2_Cl_2_ (3 mL, 60 min). Fmoc-blocked terminal amino acid (Fmoc-Gln(Trt)-OH, Fmoc-Leu-OH, Fmoc-Lys(Boc)-OH or Fmoc-Tyr(tBu)-OH) (1 mmol) and 2 mmol of NMM (220 μL) were dissolved in 3 mL of CH_2_Cl_2_ and then added to the swollen resin. The suspension was gently shaken for 2 h. Then, the resin was washed with CH_2_Cl_2_ (5 mL, 2 × 5 min), a mixture of CH_2_Cl_2_:methanol:DIPEA (17:2:1) (7 mL, 3 × 10min), and CH_2_Cl_2_ (5 mL, 2 × 5 min). The loaded resin was vacuum-dried.

#### 3.3.2. Peptide Synthesis

Peptides were synthesized following the standard Fmoc procedure. Loaded resin was swollen in CH_2_Cl_2_ (3 mL, 60 min). Then, the resin was drained, and 25% piperidine in DMF was added (5 mL, 3 × 10 min). The resin was washed with DMF (5 mL, 3 × 5 min) and CH_2_Cl_2_ (5 mL, 3 × 5 min). A mixture of 3-fold excess of Fmoc-blocked amino acid, 3-fold excess of DMT/NMM/TosO^−^, and 6-fold excess of NMM in 2 mL of DMF was added to the drained resin. The reaction time varied from 0.5 to 24 h and was controlled using the Kaiser test. After the coupling process, the resin was washed with DMF (5 mL, 3 × 5 min) and CH_2_Cl_2_ (5 mL, 3 × 5 min). The cycles were repeated to obtain the desired sequence. After the synthesis, the resin with the synthesized peptide was treated with 25% piperidine in DMF (5 mL, 3 × 10 min). The resin was washed with DMF (5 mL, 3 × 5 min) and CH_2_Cl_2_ (5 mL, 3 × 5 min) and vacuum-dried.

#### 3.3.3. Peptide Cleavage from Resin

The resin with synthesized peptide was treated with a cleavage mixture (10 mL, 4 h). The cleavage mixture used depended on the structure and consisted of either TFA/1,2-ethanedithiol/H_2_O/triisopropylsilane (94:2.5:2.5:1) or TFA/H_2_O/triisopropylsilane (95:2.5:2.5). After the reaction, the resin was filtrated and washed with CH_2_Cl_2_. The filtrate was collected and evaporated. The peptide was then precipitated in cold diethyl ether, filtered, dried, dissolved in H_2_O, and lyophilized.

- Synthesis of ^10^HDGYCLHDGVCMY^22^

Starting materials: resin (0.5 g, 0.68 mmol/g); for each coupling: Fmoc-Tyr(tBu)-OH (0.469 g, 1.02 mmol), Fmoc-Met-OH (0.379 g, 1.02 mmol), Fmoc-Cys(Trt)-OH (0.597 g, 1.02 mmol), Fmoc-Val-OH (0.346 g, 1.02 mmol), Fmoc-Gly-OH (0.303 g, 1.02 mmol), Fmoc-Asp(OtBu)-OH (0.42 g, 1.02 mmol), Fmoc-His(Trt)-OH (0.632 g, 1.02 mmol), Fmoc-Leu-OH (0.36 g, 1.02 mmol), Fmoc-Cys(Trt)-OH (0.597 g, 1.02 mmol), Fmoc-Tyr(tBu)-OH (0.469 g, 1.02 mmol), Fmoc-Gly-OH (0.303 g, 1.02 mmol), Fmoc-Asp(OtBu)-OH (0.42 g, 1.02 mmol), Fmoc-His(Trt)-OH (0.632 g, 1.02 mmol). DMT/NMM/TosO^−^ (0.421 g, 1.02 mmol) and NMM (0.224 mL, 2.04 mmol). HPLC (10–100% B in 30 min, Method 1): t_R_ = 11.8 min, purity = 91.7%. MS: 1529.5930 ([M + H]^+^, C_64_H_89_N_17_O_20_S_3_, calc. 1512.76).

- Synthesis of ^13^YCLHDGV^19^

Starting materials: resin (0.5 g, 0.88 mmol/g); for each coupling: Fmoc-Val-OH (0.448 g, 1.32 mmol), Fmoc-Gly-OH (0.392 g, 1.32 mmol), Fmoc-Asp(OtBu)-OH (0.543 g, 1.32 mmol), Fmoc-His(Trt)-OH (0.818 g, 1.32 mmol), Fmoc-Leu-OH (0.466 g, 1.32 mmol), Fmoc-Cys(Trt)-OH (0.773 g, 1.32 mmol), Fmoc-Tyr(tBu)-OH (0.607 g, 1.32 mmol). DMT/NMM/TosO^−^ (0.545 g, 1.32 mmol) and NMM (0.29 mL, 2.64 mmol). HPLC (10–100% B in 30 min, Method 1): t_R_ = 10.8 min, purity = 89.5%. MS: 806.3662 ([M + H]^+^, C_35_H_51_N_9_O_11_S, calc. 805.94).

- Synthesis of ^22^YIEALDK^28^

Starting materials: resin (0.5 g, 0.54 mmol/g); for each coupling: Fmoc-Lys(Boc)-OH (0.379 g, 0.81 mmol), Fmoc-Asp(OtBu)-OH (0.333 g, 0.81 mmol), Fmoc-Leu-OH (0.286 g, 0.81 mmol), Fmoc-Ala-OH (0.252 g, 0.81 mmol), Fmoc-Glu(OtBu)-OH (0.345 g, 0.81 mmol), Fmoc-Ile-OH (0.286 g, 0.81 mmol), Fmoc-Tyr(tBu)-OH (0.372 g, 0.81 mmol). DMT/NMM/TosO^−^ (0.335 g, 0.81 mmol) and NMM (0.178 mL, 1.62 mmol) HPLC (10–100% B in 30 min, Method 1): t_R_ = 7.4 min, purity = 83.0%. MS: 851.4854 ([M + H]^+^, C_39_H_62_N_8_O_13_, calc. 851.0).

- Synthesis of ^23^IEAL^26^

Starting materials: resin (0.5 g, 0.63 mmol/g); for each coupling: Fmoc-Leu-OH (0.334 g, 0.945 mmol), Fmoc-Ala-OH (0.294 g, 0.945 mmol), Fmoc-Glu(OtBu)-OH (0.402 g, 0.945 mmol), Fmoc-Ile-OH (0.334 g, 0.945 mmol), DMT/NMM/TosO^−^ (0.39 g, 0.945 mmol) and NMM (0.208 mL, 1.89 mmol). HPLC (10–100% B in 30 min, Method 1): t_R_ = 10.2 min, purity = 96.8%. MS: 445.2894 ([M + H]^+^, C_20_H_36_N_4_O_7_, calc. 444.55).

- Synthesis of ^34^VVGYIGERCQYRDL^47^

Starting materials: resin (0.5 g, 0.7 mmol/g); for each coupling: Fmoc-Leu-OH (0.371 g, 1.05 mmol), Fmoc-Asp(OtBu)-OH (0.432 g, 1.05 mmol), Fmoc-Arg(Pbf)-OH (0.681 g, 1.05 mmol), Fmoc-Tyr(tBu)-OH (0.483 g, 1.05 mmol), Fmoc-Gln(Trt)-OH (0.641 g, 1.05 mmol), Fmoc-Cys(Trt)-OH (0.615 g, 1.05 mmol), Fmoc-Arg(Pbf)-OH (0.681 g, 1.05 mmol), Fmoc-Glu(OtBu)-OH (0.447 g, 1.05 mmol), Fmoc-Gly-OH (0.312 g, 1.05 mmol), Fmoc-Ile-OH (0.371 g, 1.05 mmol), Fmoc-Tyr(tBu)-OH (0.483 g, 1.05 mmol), Fmoc-Gly-OH (0.312 g, 1.05 mmol), Fmoc-Val-OH (0.356 g, 1.05 mmol), Fmoc-Val-OH (0.356 g, 1.05 mmol). DMT/NMM/TosO^−^ (0.39 g, 1.05 mmol) and NMM (0.231 mL, 2.1 mmol). HPLC (10–100% B in 30 min, Method 1): t_R_ = 12.2 min, purity = 87.7%. MS: 835.9354 ([M + H]^2+^, C_73_H_115_N_21_O_22_S, calc. 1670.98).

- Synthesis of ^38^IGERCQ^43^

Starting materials: resin (0.5 g, mmol/g); for each coupling: Fmoc-Gln(Trt)-OH (0.632 g, 1.035 mmol), Fmoc-Cys(Trt)-OH (0.606 g, 1.035 mmol), Fmoc-Arg(Pbf)-OH (0.672 g, 1.035 mmol), Fmoc-Glu(OtBu)-OH (0.44 g, 1.035 mmol), Fmoc-Gly-OH (0.308 g, 1.035 mmol), Fmoc-Ile-OH (0.366 g, 1.035 mmol). DMT/NMM/TosO^−^ (0.427 g, 1.035 mmol) and NMM (0.228 mL, 2.07 mmol). HPLC (10–100% B in 30 min, Method 1): t_R_ = 8.3 min, purity = 90%. MS: 705.3667 ([M + H]^+^, C_27_H_48_N_10_O_10_S, calc. 704.83).

- Synthesis of ^26^LDKYACNCV^34^

Starting materials: resin (0.5 g, 0.73 mmol/g); for each coupling: Fmoc-Val-OH (0.372 g, 1.095 mmol), Fmoc-Cys(Trt)-OH (0.641 g, 1.095 mmol), Fmoc-Asn(Trt)-OH (0.653 g, 1.095 mmol), Fmoc-Cys(Trt)-OH (0.641 g, 1.095 mmol), Fmoc-Ala-OH (0.341 g, 1.095 mmol), Fmoc-Tyr(tBu)-OH (0.503 g, 1.095 mmol), Fmoc-Lys(Boc)-OH (0.513 g, 1.095 mmol), Fmoc-Asp(OtBu)-OH (0.451 g, 1.095 mmol), Fmoc-Leu-OH (0.387 g, 1.095 mmol), DMT/NMM/TosO^−^ (0.452 g, 1.095 mmol) and NMM (0.241 mL, 2.19 mmol). HPLC (10–100% B in 30 min, Method 1): t_R_ = 11.6 min, purity = 79.2%. MS: 1028.4756 ([M + H]^+^, C_43_H_69_N_11_O_14_S_2_, calc. 1028.24).

- Synthesis of ^10^HDGYCL^15^

Starting materials: resin (0.5 g, 0.63 mmol/g); for each coupling: Fmoc-Leu-OH (0.334 g, 0.945 mmol), Fmoc-Cys(Trt)-OH (0.553 g, 0.945 mmol), Fmoc-Tyr(tBu)-OH (0.434 g, 0.945 mmol), Fmoc-Gly-OH (0.281 g, 0.945 mmol), Fmoc-Asp(OtBu)-OH (0.389 g, 0.945 mmol), Fmoc-His(Trt)-OH (0.586 g, 0.945 mmol). DMT/NMM/TosO^−^ (0.39 g, 0.945 mmol) and NMM (0.208 mL, 1.89 mmol). HPLC (10–100% B in 30 min, Method 1): t_R_ = 11.7 min, purity = 84.5%. MS: 707.3398 ([M + H]^+^, C_30_H_42_N_8_O_10_S, calc. 706.81).

- Synthesis of ^10^HDGYCL^15^+^29^Y+^41^R

Starting materials: resin (0.5 g, 0.52 mmol/g); for each coupling: Fmoc-Arg(Pbf)-OH (0.506 g, 0.78 mmol), Fmoc-Tyr(tBu)-OH (0.358 g, 0.78 mmol), Fmoc-Leu-OH (0.276 g, 0.78 mmol), Fmoc-Cys(Trt)-OH (0.457 g, 0.78 mmol), Fmoc-Tyr(tBu)-OH (0.358 g, 0.78 mmol), Fmoc-Gly-OH (0.232 g, 0.78 mmol), Fmoc-Asp(OtBu)-OH (0.321 g, 0.78 mmol), Fmoc-His(Trt)-OH (0.483 g, 0.78 mmol). DMT/NMM/TosO^−^ (0.322 g, 0.78 mmol) and NMM (0.172 mL, 1.56 mmol). HPLC (10–100% B in 30 min, Method 1): t_R_ = 11.0 min, purity = 81.5%. MS: 1026.5228 ([M + H]^+^, C_45_H_63_N_13_O_13_S, calc. 1026.17).

- Synthesis of ^43^QYRDL^47^

Starting materials: resin (0.5 g, 0.60 mmol/g); for each coupling: Fmoc-Leu-OH (0.318 g, 0.9 mmol), Fmoc-Asp(OtBu)-OH (0.37 g, 0.9 mmol), Fmoc-Arg(Pbf)-OH (0.584 g, 0.9 mmol), Fmoc-Tyr(tBu)-OH (0.414 g, 0.9 mmol), Fmoc-Gln(Trt)-OH (0.55 g, 0.9 mmol). DMT/NMM/TosO^−^ (0.372 g, 0.9 mmol) and NMM (0.198 mL, 1.8 mmol). HPLC (10–100% B in 30 min, Method 1): t_R_ = 10.0 min, purity = 75.1%. MS: 694.4089 ([M + H]^+^, C_30_H_47_N_9_O_10_, calc. 693.78).

### 3.4. MST Analysis

#### 3.4.1. Protein Labeling

Recombinant human EGFR protein (Active) (ab155639) was reconstituted with 500 μL of 0.45 μm filtered distilled water. Then, 100 nM solution of EGFR was prepared by mixing 3.6 μL of protein with 96.4 μL PBS-T (PBS with 0.05% Tween20). RED-tris-NTA 2nd Generation (5 μM) was obtained by dissolving 250 pM of dye in 50 μL PBS-T. A solution of dye in PBS-T (50 nM) was prepared by mixing 1 μL of dye with 99 μL PBS-T. A solution of the protein (95 μL) was mixed with the solution of dye (95 μL) and incubated at room temperature for 30 min. The sample was then centrifuged for 10 min at 4 °C. The final labeled protein concentration in the assay was 25 nM.

#### 3.4.2. Binding Assay

A 2× concentration of ligand (EGF fragments) was prepared in PBS-T. First, 10 μL of PBS-T was added to 16 PCR tubes. Then, 20 μL of the ligand was transferred to PCR tube 1. Subsequently, 10 μL of the solution was transferred to PCR tube 2 and mixed by pipetting; 10 μL of the solution from PCR tube 2 was transferred to PCR tube 3 and mixed by pipetting; this procedure was repeated for PCR tubes 4–16. From PCR tube 16, 10 μL of the solution was discarded. Finally, 10 μL of labeled EGFR was added to each PCR tube and mixed by pipetting. The samples were incubated for 30 min at room temperature prior to MST assays. Analyses were performed using NanoTemper Technologies (München, Germany).

### 3.5. Biological Studies

Primary Dermal Fibroblast (ATCC, Manassas, USA) was grown in Fibroblast Basal Media supplemented with Fibroblast Growth Kit components (ATCC, Manassas, VA, USA). The cells were cultured under standard conditions (37 °C, humidified atmosphere of 5% CO_2_ in air). The medium was replaced every 2–3 days (75% confluence). Cells between the third and fifth passages were used in the experiment.

#### 3.5.1. Cell Viability

Cell viability was estimated using a resazurin-based in vitro toxicology assay kit, which is a type of colorimetric test. It contains the key compound resazurin as an indicator of redox. The amount of red intermediate pigment (resorufin) that is obtained from resazurin reduction is directly proportional to the number of viable cells. Due to the high sensitivity of this assay, it is ideally suited for measuring the cytotoxicity of compounds. Cells were plated and incubated in 96-well plates (8 × 10^3^ cells per well). To assess the cytotoxicity of the compounds, a number of dilutions were made: 10, 100, and 1000 ng/mL. The cells were then incubated with the compounds for 1 day and 7 days. Untreated cells were used as a negative control. Cells incubated with 1.5% DMSO were used as a positive control. Following incubation of the cells, the well contents were removed, and the wells were rinsed with 1× DPBS. Next, 100 μL of the resazurin solution (10% of cell culture medium volume) was added to each well and incubated with the resazurin solution for 2 h. Absorbance was measured at a wavelength of 600 nm and at a reference wavelength of 690 nm using a Synergy HT (BioTek) (Hampton, NH, USA) spectrophotometer [105].

Cell viability was calculated using the following formula:

$$\mathrm{Cell}\;\mathrm{viability}\;(\%)=\frac{A_{sample}\left(A_{600}-A_{690}\right)-A_{blank}\left(A_{600}-A_{690}\right)}{A_{control}\left(A_{600}-A_{690}\right)-A_{blank}\left(A_{600}-A_{690}\right)}\times 100$$

#### 3.5.2. Live/Dead Test

To evaluate proliferation and cytotoxicity, a “live/dead” test (Viability/Cytotoxicity Kit, Molecular Probes) was used. Cells were seeded at 6 × 104 cells/mL/well/sample in 2 mL of Fibroblast Basal Media supplemented with Fibroblast Growth Kit components (ATCC, Manassas, VA, USA). After 24 h, the medium was completely replaced. Cells in the positive control and negative control were cultured in medium containing FGF. In the remaining samples, media without FGF and with the addition of heat-inactivated serum were used. Recombinant human EGF protein (Abcam, Cambridge, UK) was added to the EGF sample at a final concentration of 100 ng/mL. The EGF protein was also used at a final concentration of 100 ng/mL with samples 10–22, 13–19, 22–28, 26–34, 10–15, and 10–15a. During the experiment, half of the medium was changed every 2 days. Proliferation and cytotoxicity were evaluated after 1 day and after 7 days of culturing cells in the presence of EGF. After that time, a mixture of two fluorescent dyes was used: Calcein-AM for the staining of living cells and ethidium bromide for the labeling of dead cells.

The samples were examined using an Olympus GX 71 fluorescence microscope equipped with a digital camera (DP70) (Olympus, Evident, Tokyo, Japan). The results were submitted to one-way ANOVA analysis. If the *p* value was less than 0.05, the results were considered significantly different.

## 4. Conclusions

The results of this study show that specific polyclonal antibodies can be used to reconstruct the outer sphere of the growth factor and to find EGF fragments that are involved in binding with the receptor. The results support those of a previous study conducted by Ogiso et al. Epitopes occurring in nature are mostly discontinuous. However, our experiment successfully identified linear fragments composed or partly composed of discontinuous epitopes. Importantly, the sequences that we found comprise all or almost all amino acids that take part in binding with the receptor at particular sites.

The presented method can be used to search not only for full sequences but also for the shortest fragment that binds to the receptor. This could simplify the beginning of the process of designing novel peptides capable of activating the receptor. MST binding tests showed that selected fragments found by immunoassays bind to EGFR. Shortening the active fragment may help to maintain binding activity. Even more importantly, the selected fragments imitate the structure of the native protein. The peptide fragments are natural constituents of polypeptide, which is widespread in the human body. This means that they should be well tolerated in in vitro or in vivo studies, as suggested by cytotoxicity assays. Based on the results presented in this paper, fragments ^10^HDGYCLHDGVCMY^22^, ^34^VVGYIGERCQYRDL^47^, ^38^IGERCQ^43^, and ^43^QYRDL^47^ are promising peptides for therapeutic applications as short chains able to bind with EGFR. The presence of essential amino acids ensures that these fragments can maintain their native conformation binding to the specified protein. Moreover, the inclusion of amino acids that do not take part in receptor binding opens a wide range of opportunities for modifying peptides (substituting them for other amino acid residues or incorporating new side-chain groups) to obtain desired properties (e.g., strengthening binding affinity) for use in different therapies.

## Figures and Tables

**Table 1:** Sequences of each synthesized EGF fragment attached to the cellulose matrix. Division of peptides in terms of their ability to interact with polyclonal antibodies: very strong: ++; strong: +; moderate: +/−; no ability to interact: −. The staining intensities of antibody–EGF fragment complexes are also given.

Position	Intensity (Staining Value)	Sequence	Position	Intensity (Staining Value)	Sequence
A1	− (14.8)	H-^1^NSDSECPLSH^10^-*matrix*	B3	+/− (63.8)	H-^23^IEALDKYACN^32^-*matrix*
A2	− (22.3)	H-^2^SDSECPLSHD^11^-*matrix*	B4	− (30.5)	H-^24^EALDKYACNC^33^-*matrix*
A3	+/− (71.7)	H-^3^DSECPLSHDG^12^-*matrix*	B5	− (29.6)	H-^25^ALDKYACNCV^34^-*matrix*
A4	+/− (55.2)	H-^4^SECPLSHDGY^13^-*matrix*	B6	+/− (46.6)	H-^26^LDKYACNCVV^35^-*matrix*
A5	+/− (64.5)	H-^5^ECPLSHDGYC^14^-*matrix*	B7	+/− (55.3)	H-^27^DKYACNCVVG^36^-*matrix*
A6	+/− (58.4)	H-^6^CPLSHDGYCL^15^-*matrix*	B8	+/− (63.5)	H-^28^KYACNCVVGY^37^-*matrix*
A7	+/− (62.2)	H-^7^PLSHDGYCLH^16^-*matrix*	B9	+ (91.1)	H-^29^YACNCVVGYI^38^-*matrix*
A8	+/− (59.3)	H-^8^LSHDGYCLHD^17^-*matrix*	B10	+/− (58.5)	H-^30^ACNCVVGYIG^39^-*matrix*
A9	+/− (63.8)	H-^9^SHDGYCLHDG^18^-*matrix*	B11	+/− (60.3)	H-^31^CNCVVGYIGE^40^-*matrix*
A10	+ (91.3)	H-^10^HDGYCLHDGV^19^-*matrix*	B12	+/− (56.3)	H-^32^NCVVGYIGER^41^-*matrix*
A11	+ (90.5)	H-^11^DGYCLHDGVC^20^-*matrix*	B13	+/− (65.7)	H-^33^CVVGYIGERC^42^-*matrix*
A12	+ (92.4)	H-^12^GYCLHDGVCM^21^-*matrix*	B14	+/− (69.4)	H-^34^VVGYIGERCQ^43^-*matrix*
A13	+ (93.0)	H-^13^YCLHDGVCMY^22^-*matrix*	B15	+ (91.0)	H-^35^VGYIGERCQY^44^-*matrix*
A14	− (31.6)	H-^14^CLHDGVCMYI^23^-*matrix*	B16	+ (98.0)	H-^36^GYIGERCQYR^45^-*matrix*
A15	− (41.6)	H-^15^LHDGVCMYIE^24^-*matrix*	B17	+ (106.2)	H-^37^YIGERCQYRD^46^-*matrix*
A16	− (44.8)	H-^16^HDGVCMYIEA^25^-*matrix*	B18	+/− (76.1)	H-^38^IGERCQYRDL^47^-*matrix*
A17	+/− (88.9)	H-^17^DGVCMYIEAL^26^-*matrix*	B19	− (28.9)	H-^39^GERCQYRDLK^48^-*matrix*
A18	+ (116.0)	H-^18^GVCMYIEALD^27^-*matrix*	B20	+/− (87.3)	H-^40^ERCQYRDLKW^49^-*matrix*
A19	++ (128.0)	H-^19^VCMYIEALDK^28^-*matrix*	C1	+ (91.5)	H-^41^RCQYRDLKWW^50^-*matrix*
A20	++ (132.8)	H-^20^CMYIEALDKY^29^-*matrix*	C2	+ (91.4)	H-^42^CQYRDLKWWE^51^-*matrix*
B1	++ (130.3)	H-^21^MYIEALDKYA^30^-*matrix*	C3	+ (99.4)	H-^43^QYRDLKWWEL^52^-*matrix*
B2	++ (122.4)	H-^22^YIEALDKYAC^31^-*matrix*	C4	+ (104.2)	H-^44^YRDLKWWELR^53^-*matrix*

**Table 2:** Sequences involved in binding with EGFR and peptides found via interactions with antibodies. Residues that take part directly in binding with the receptor according to Ogiso et al. [91] are marked in red.

	From the Literature	Found
fragment 1	^18^GVCMYIEALDKYACNC^33^	^17^DGVCMYIEALDKYACN^32^
		^27^DKYACNCVVGYIG^39^
fragment 2	^10^HDGYCL^15^ + ^29^Y + ^41^R	^1^NSDSECPLSHDGYCLHDGVCMY^22^
fragment 3	^43^QYRDL^47^	^33^CVVGYIGERCQYRDL^47^
		^40^ERCQYRDLKWWELR^53^

**Table 3:** Structures of EGF fragments synthesized in the solid phase and their characteristics.

Fragment	Number of Amino Acids	Purity (%) Based on HPLC	Molar Weight (g/mol)	Measured *m*/*z*
^10^HDGYCLHDGVCMY^22^	13	91.7	1512.72	510.2 (3+)
				764.8 (2+)
				1529.6 (+) (product with oxidized Met)
^13^YCLHDGV^19^	7	89.5	805.91	403.7 (2+)
				806.4 (+)
^22^YIEALDK^28^	7	83.0	850.97	426.3 (2+)
				851.5 (+)
^23^IEAL^26^	4	96.8 dimer	444.53	445.3 (+)
^34^VVGYIGERCQYRDL^47^	14	87.7	1670.92	557.6 (3+)
				835.9 (2+)
^38^IGERCQ^43^	6	90	704.81	353.2 (2+)
				705.4 (+)
^26^LDKYACNCV^34^	9	79.2	1028.22	514.7 (2+)
				1028.5 (+)
^10^HDGYCL^15^	6	84.5	706.27	707.3 (+)
^10^HDGYCL^15^+^29^Y+^41^R	8	81.5	1025.44	513.8 (2+)
				1026.5 (+)
^43^QYRDL^47^	5	75.1	693.35	347.7 (2+)
				694.4 (+)

**Table 4:** Results of MST experiments of binding of EGF fragments to EGFR, with various concentrations of EGF fragments. The labeled EGFR concentration was 25 nM.

Fragment	Assay Concentration	Kd
10–22	50 μM	70.5 nM
	100 μM	8.2 μM
	463 μM	21.2 μM
	1 mM	35.9 μM
	3 mM	416 μM
10–15	50 μM	66.4 nM
	100 μM	17.4 nM
	250 μM	48.4 μM
	2.5 mM	431 μM
	5 mM	663 μM
	6.02 mM	715 μM
	10 mM	533 μM
22–28	10 μM	-
	250 μM	4.02 nM
	500 μM	-
	3 mM	1.96 mM
	5 mM	1.31 mM
	10 mM	1.65 mM
23–26	1.25 μM	-
	2.5 μM	-
	50 μM	-
	100 μM	-
	5 mM	-
	10 mM	5.09 mM
34–47	50 μM	5.92 μM
	141 μM	4.62 μM
	350 μM	1.21 μM
	705 μM	2.17 μM
	1 mM	530 nM
38–43	10 μM	709 nM
	500 μM	154 nM
	2.5 mM	108 nM
	5 mM	157 nM
43–47	500 μM	4.93 μM
	10 mM	125 μM
EGF	500 nM	10.6 nM

## Data Availability

Data are contained within the article and supplementary materials.

## Supplementary Materials

The supporting information can be downloaded at https://www.mdpi.com/article/10.3390/ijms25031470/s1.
