# Supplementary material for: Searching for EGF Fragments Recreating the Outer Sphere of the Growth Factor Involved in Receptor Interactions
Source: Int J Mol Sci. 2024 Jan 25;25(3):1470. doi: 10.3390/ijms25031470 (PMC10855902; doi:10.3390/ijms25031470)
Supplement: Supplementary file 1 [file ijms-25-01470-s001.zip › ijms-2795965-supplementary.pdf]

Searching for EGF fragments recreating the outer sphere growth factor involved in receptor interactions

Katarzyna Czerczak-Kwiatkowska[1], Marta Kamiska[2], Justyna Fraczyk[1], Ireneusz Majsterek[3], Beata Kolesinska[1]*

1. Institute of Organic Chemistry, Faculty of Chemistry, Lodz University of Technology, Zeromskiego 116, 90-924 Lodz, Poland; katarzyna.czerczak@dokt.p.lodz.pl (K.C-K.); justyna.fraczyk@p.lodz.pl (J.F.); beata.kolesinska@p.lodz.pl (B.K.)

2. Division of Biophysics, Institute of Materials Science and Engineering, Lodz University of Technology, Stefanowskiego 1/15, 90-924 Lodz, Poland; marta.kaminska@p.lodz.pl (M.K.)

3. Department of Clinical Chemistry and Biochemistry, Medical University of Lodz, Narutowicza 60, 90-136 Lodz, Poland; ireneusz.majsterek@umed.lodz.pl (I.M.)

Modification of cellulose substrate - incorporation of a triazine linker immobilized on cellulose

A procedure previously developed by us was used to obtain the isocyanurate derivative of amino acid attached to cellulose [1].

Immobilization of 2,4-dichloro-6-methoxy-1,3,5-triazine (DCMT) on cellulose

Whatman 1 CHR cellulose sheets (4 pieces) on dimensions 10 x 15 cm, were treated with 1.0 M aq. NaOH (500 mL) for 30 min. The base solution was then removed, and the cellulose sheets were dried with a paper towel. In the next step, they were treated with 1.2 M solution of 2,4-dichloro-6-methoxy-1,3,5-triazine (DCMT) (111 g, 0.6 mol) in THF (500 mL). Solid, finely powdered $NaHCO_3$ (50.4 g, 0.6 mol) and diisopropylamine (DIPEA) (108 mL, 0.6 mol) were added to the solution. After 3 hours the solution was removed, and the cellulose was washed sequentially with THF (3 x 150 mL), acetone (3 x 150 mL), and CH_2Cl_2 (3 x 150 mL) and allowed to air dry.

Functionalization of DCMT-modified cellulose with N-methylmorpholine (NMM)

DCMT-modified cellulose sheets were treated with a solution of N-methylmorpholine (NMM) in THF (v: v 1: 1) (200 mL). The reactions were carried out for 30 minutes by vigorously shaking the mixture. The NMM solution was then removed and the cellulose sheets were washed with THF (2 x 150 mL, 5 min) and dried with a paper towel.

C-terminal Fmoc-glycine incorporation (synthesis of Fmoc-Gly triazine ester immobilized on cellulose)

Cellulose sheets functionalized with DCMT and N-methylmorpholine (NMM) (4 pieces) were treated with Fmoc-Gly-OH (4.46 g, 15 mmol), (825 µL, 7.5 mmol) in 100 mL THF : dichloromethane (DCM) (1 : 1). The reaction was carried out for 1.5 h with gentle shaking.
The solution was then removed, the cellulose sheets were washed with DMF (2 x 150 mL) for 5 min and CH_2Cl_2 (2 x 150 mL) for 5 min and then dried with a paper towel.

Thermal rearrangement of the Fmoc-Gly triazine ester immobilized on cellulose

The dried Fmoc-Gly triazine ester modified cellulose sheets were heated in refluxing toluene for 8 h. The solvent was then removed, the sheets were dried with a paper towel and dried in a vacuum desiccator until constant weight.

Acetylation of a cellulose matrix modified with an isocyanurate derivative of Fmoc-Gly

The cellulose sheets were treated with a 10% solution of acetic anhydride (5 mL, 0.088 mol) in DMF (35 mL) in the presence of a 20% solution of diisopropylethylamine (10 mL, 0.057 mmol). After 30 min of gentle shaking, the acetylation mixture was removed, and the cellulose sheets were washed with DMF (2 x 50 mL) 5 min, CH_2Cl_2 (2 x 50 mL) 5, min, and C_2H_5OH (3 x 50 mL) 5 min and again

CH_2Cl_2 (2 x 50 mL)) 5 min. The solvent was then removed, and the sheets were dried with a paper towel and dried in a vacuum desiccator until constant weight.

Fmoc deprotection from the C-terminal glycine residue immobilized on cellulose

The Fmoc group from the amine glycine function was removed by treatment with 25% piperidine in DMF (40 mL). The cellulose sheets immersed in the solution were gently shaken for 30 min, then the solution was removed, the cellulose sheets were washed in 5-minute cycles with DMF (3 x 40 mL). The synthesis scheme is shown in Figure S1.

a

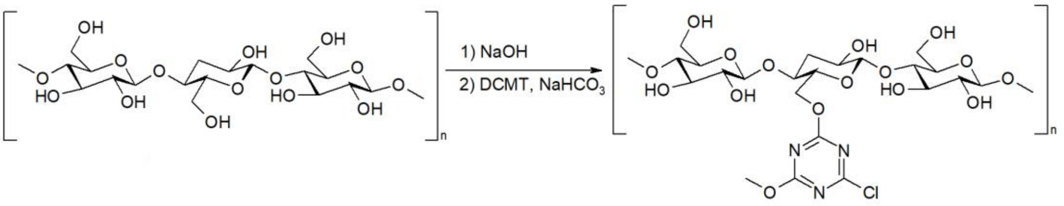
1) NaOH
2) DCMT, NaHCO3

b

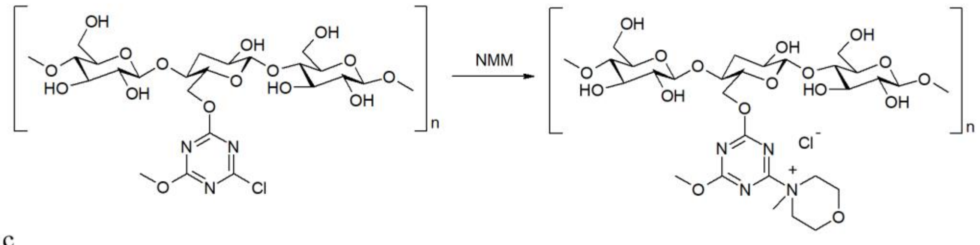
NMM

c

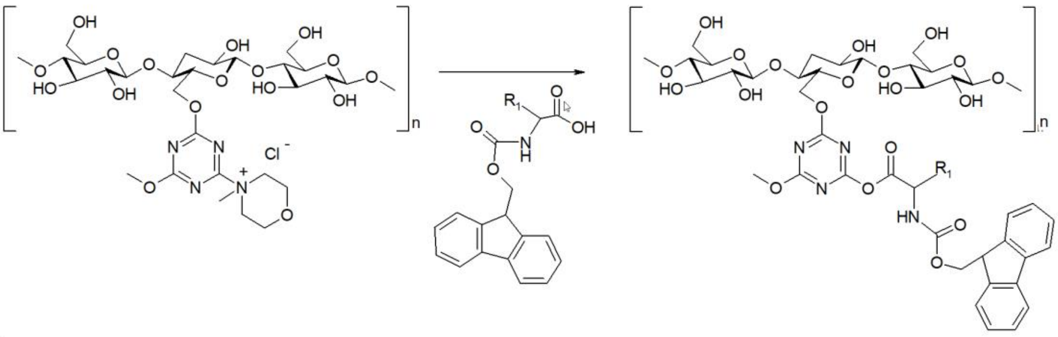

d

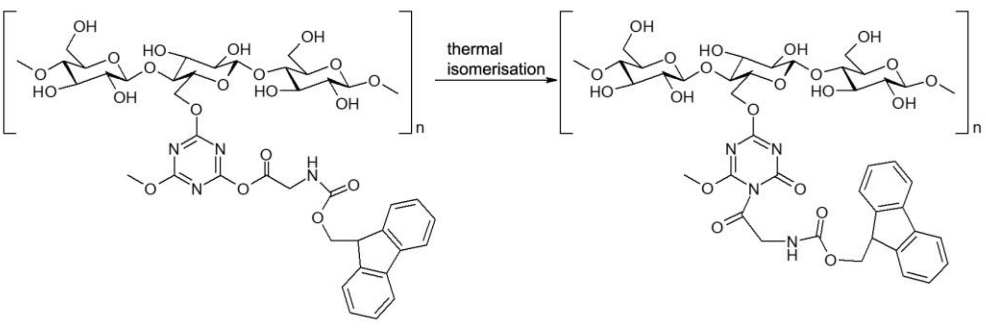
thermal
isomerisation

e

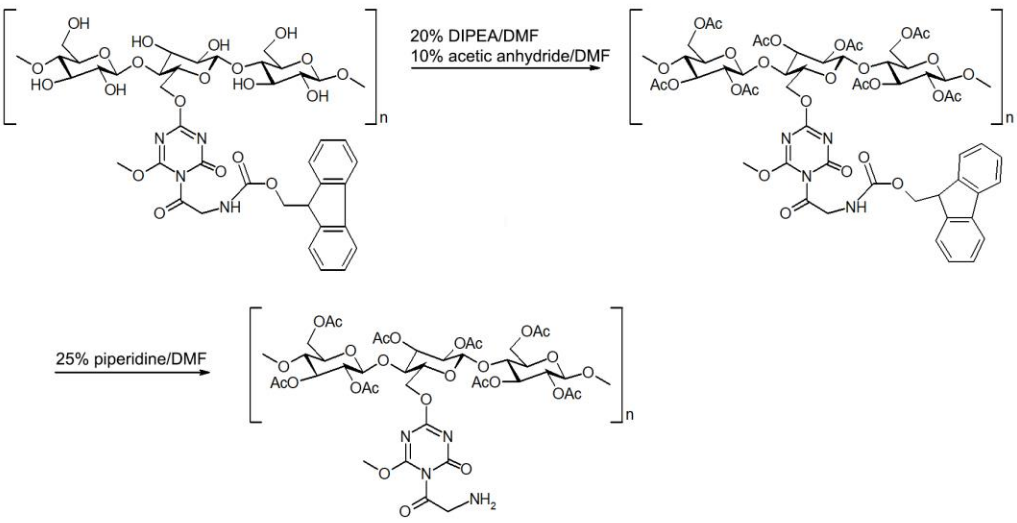
20% DIPEA/DMF
10% acetic anhydride/DMF
25% piperidine/DMF

Figure S1. Cellulose functionalization resulting in a cellulose matrix useful in SPOT synthesis of peptide library.

The obtained modified cellulose was used in the automatic SPOT synthesis [1]. The synthesis was performed using a synthetic protocol developed by us using triazine condensing reagents [2].

Automatic SPOT synthesis of the library of EGF fragments

Detailed protocols and reports from Intavis' SPOT ResPep SL automatic synthesizer are presented below.

Incorporation of the first amino acid. Report on the work of the automatic synthesizer

```
Prepare
   1 Memo            ResPep SL version, SPOT method
   2 RinseNeedle     1000 / 1500 ul
   3 WashMembrane    1500 µl, solvent - 2,  2x
   4 Extract         720 s
   ...
Cycle :  1 -> 1
   5 PreActivate     0.15+0.073+0.003+0.17->Peptides
   6 Coupling        (PreActivate)->Peptides
   7 Coupling        (PreActivate)->Peptides
   8 RinseNeedle     500 / 1500 ul
   9 Extract         120 s
   10 PreActivate    0.15+0.073+0.003+0.17->Peptides
   11 Coupling       (PreActivate)->Peptides
   12 Coupling       (PreActivate)->Peptides
   13 RinseNeedle    500 / 1500 ul
   14 WashMembrane   1500 µl, solvent - 1,  4x
   15 WashMembrane   1500 µl, solvent - 2,  2x
   16 Extract        60 s
   17 RinseNeedle    500 / 1500 ul
```

The synthesis was programmed according to the following procedure:

```
Activator                        76.8
Base                             40.5
NMP          NMP                  7.5
Piperidine   Piperidine 20%     468.8    93.8 ml

  reagents weight calculation:
    3 % relative  +  6000 µl absolute excess

    Piperidine:                    c = 0 %

  Notes:
HOBt                            135.12 g/mol
HOBt . H2O   153.12 g/mol
HOAt                            136.11 g/mol
Oxyma Pure   142.10 g/mol
all stocks in DMF or NMP @ a 1.5 M solution

DIC          MW 126,2           d=0,815
1.1M solution ~ 17.033 %


Solvents used :
   Dilutor-1
Reservoir    DMF                   1336

   Dilutor-2
Solvent-1    solvent 1             1692
Solvent-2    solvent 2              720
```

For a better visualization of the automatic SPOT synthesis procedure, the figures below show reports on the individual stages of cycle 1.

Cycle 1, Step 5 - Pre-Activation:

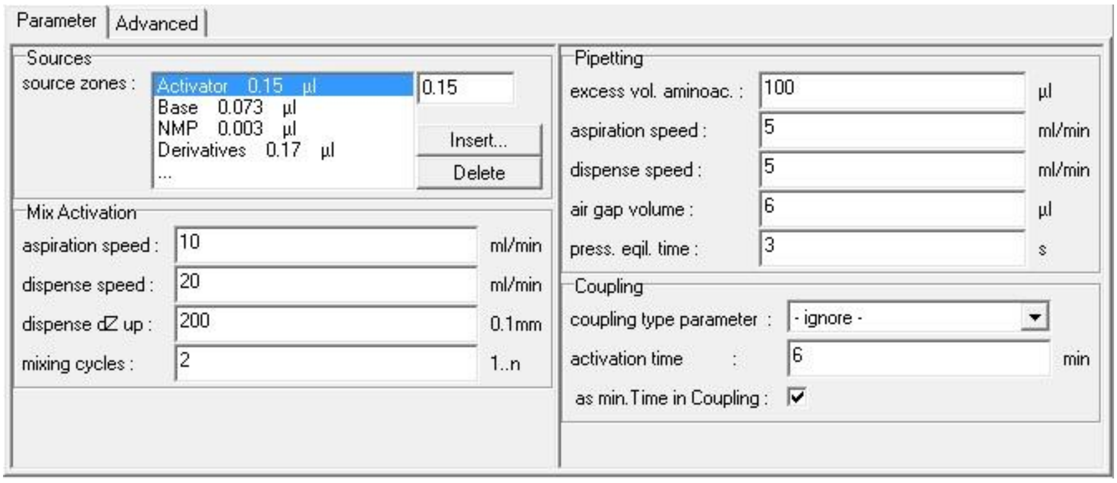

Cycle 1, step 6, 7- Coupling:

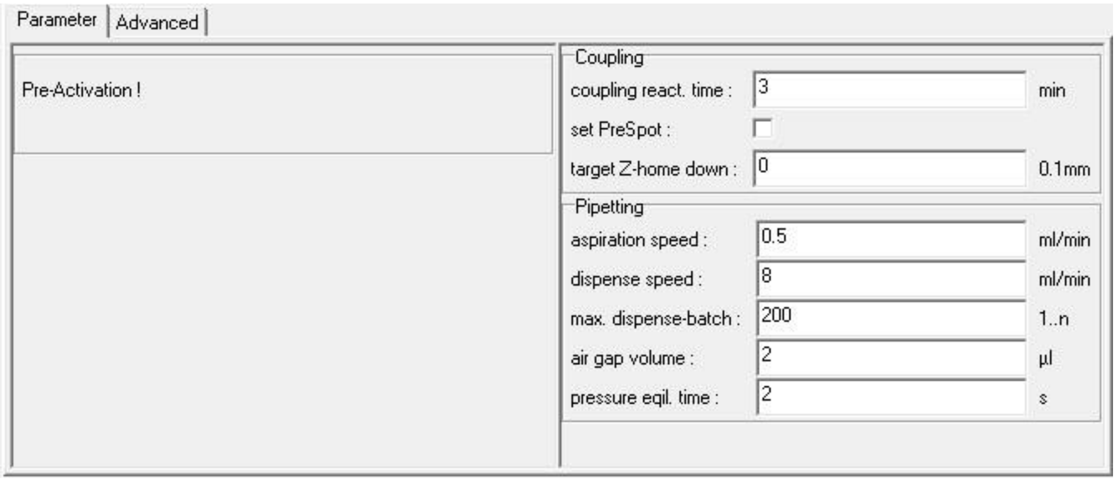

Cycle 1, step 8 - Washing the needle:

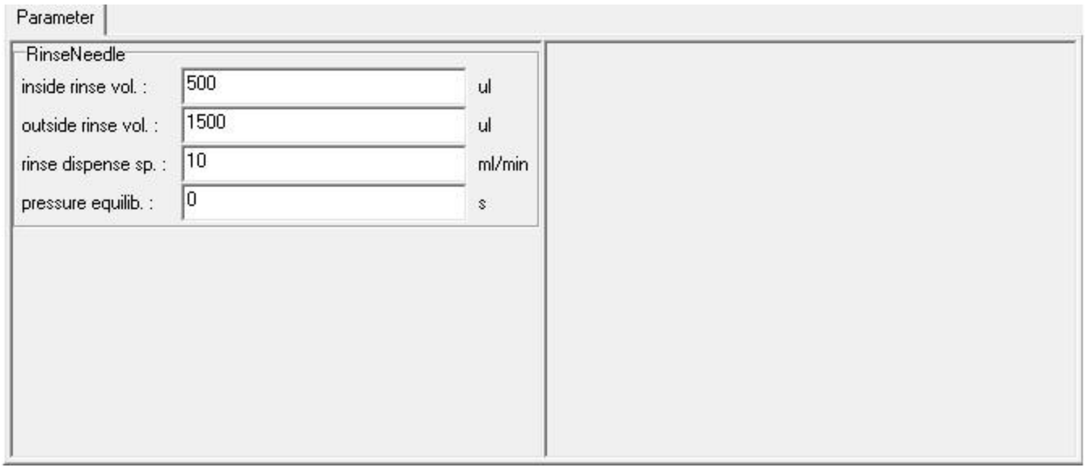

Cycle 1, step 14, 15 - Washing the membrane:

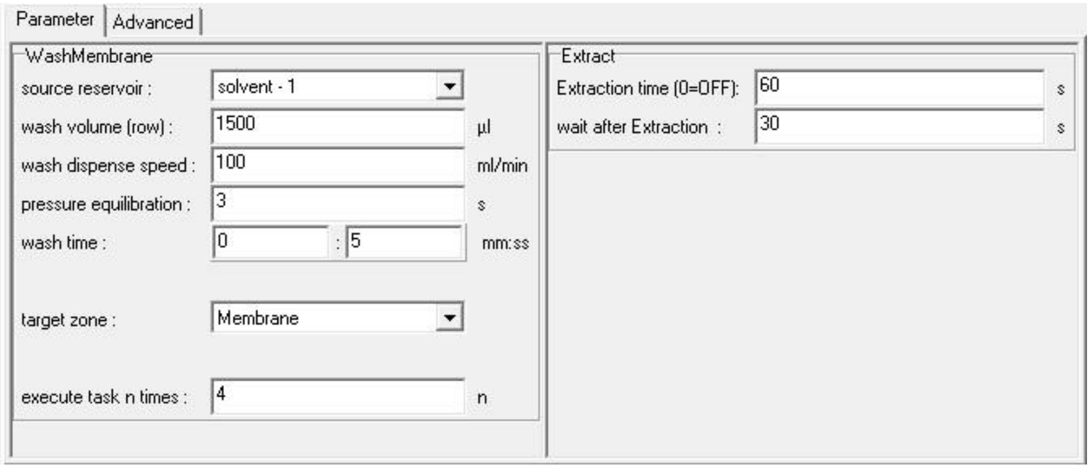

Cycle 1, step 16 - Membrane drying:

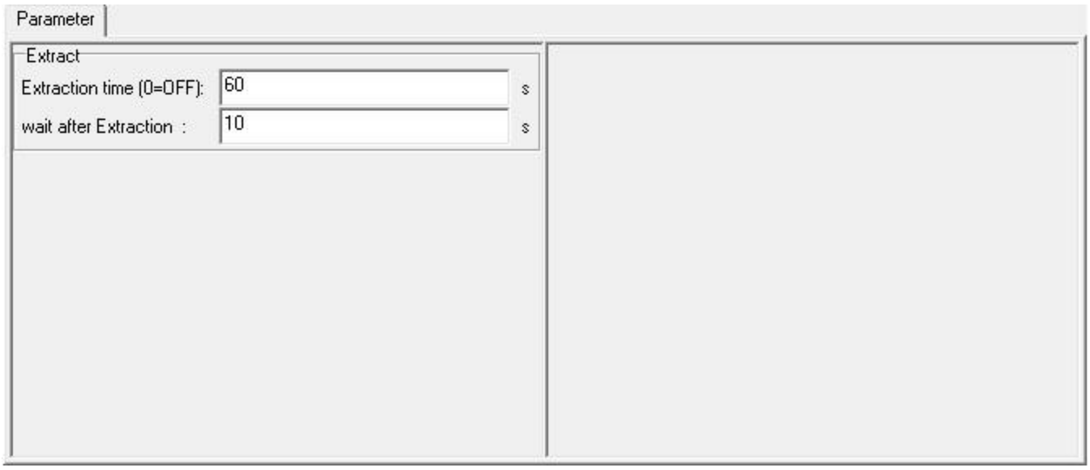

Incorporation of the second amino acid. Report on the work of the automatic synthesizer

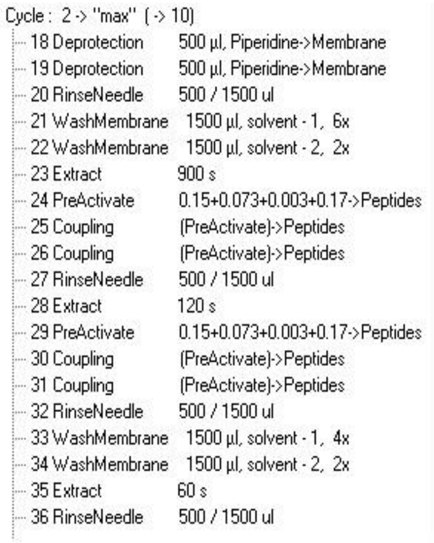

In cycle 2, the additional stage that started this cycle was the deprotection of the Fmoc group. To better illustrate the parameters of the procedure of this stage, the report generated by the synthesizer is presented below. The incorporation of subsequent amino acids was analogous to the procedure described in cycle 2.

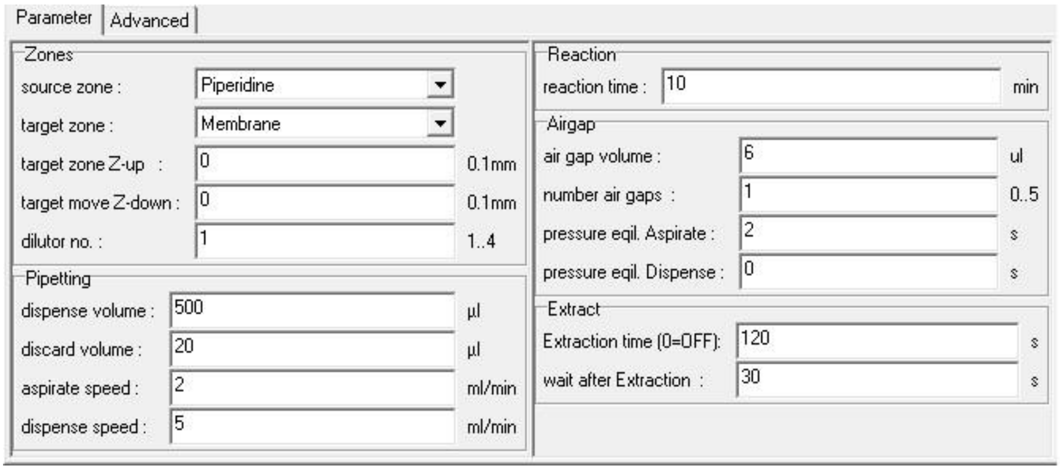

Figure S2. Synthetic protocols generated during automatic SPOT synthesis using from Intavis' SPOT ResPep SL automatic synthesizer.

Acquisition Parameter

Source Type	ESI	Ion Polarity	Positive	Set Nebulizer	2.4 Bar
Focus	Not active	Set Capillary	4500 V	Set Dry Heater	250 °C
Scan Begin	50 m/z	Set End Plate Offset	-500 V	Set Dry Gas	6.0 l/min
Scan End	3000 m/z	Set Collision Cell RF	600.0 Vpp	Set Divert Valve	Waste

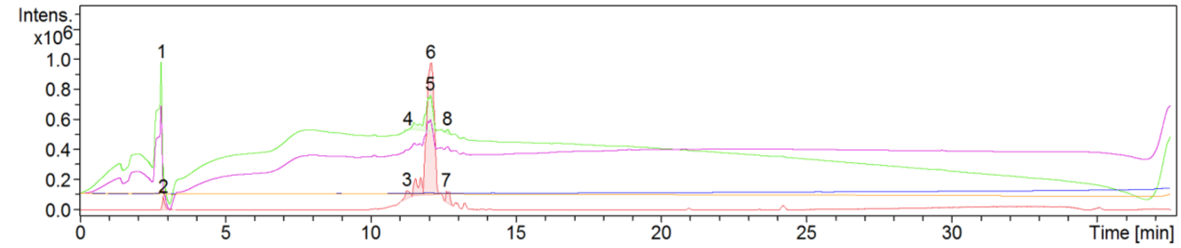

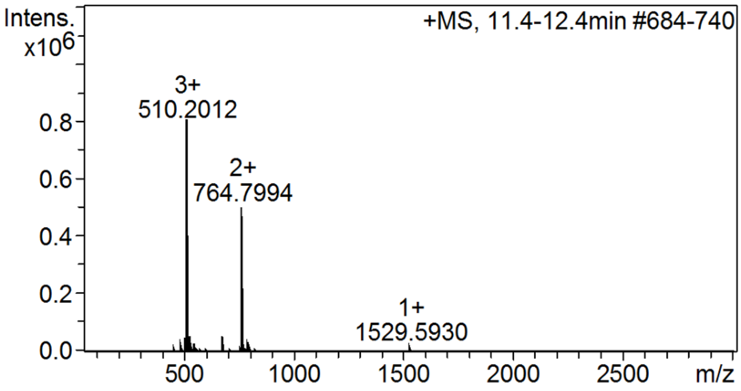

Figure S3. LC-MS spectrum of [10]HDGYCLHDGVCMY[22] fragment.

Acquisition Parameter

Source Type	ESI	Ion Polarity	Positive	Set Nebulizer	2.4 Bar
Focus	Not active	Set Capillary	4500 V	Set Dry Heater	250 °C
Scan Begin	50 m/z	Set End Plate Offset	-500 V	Set Dry Gas	6.0 l/min
Scan End	3000 m/z	Set Collision Cell RF	600.0 Vpp	Set Divert Valve	Waste

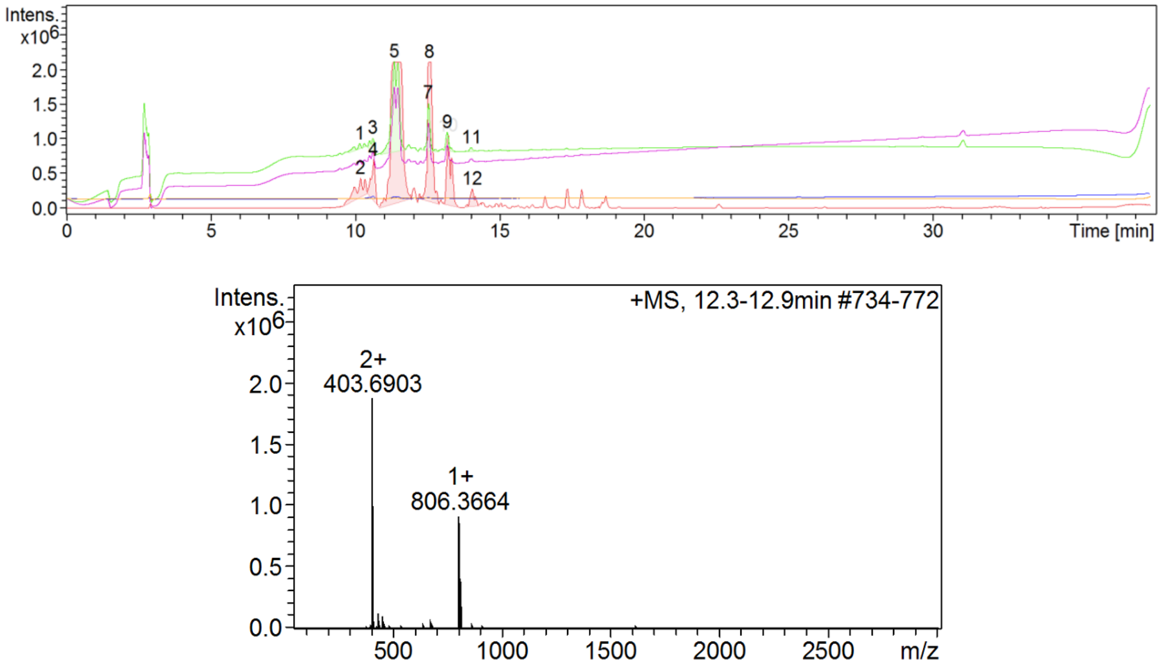

Figure S4. LC-MS spectrum of [13]YCLHDGV[19] fragment.

Acquisition Parameter

Source Type	ESI	Ion Polarity	Positive	Set Nebulizer	2.4 Bar
Focus	Not active	Set Capillary	4500 V	Set Dry Heater	250 °C
Scan Begin	50 m/z	Set End Plate Offset	-500 V	Set Dry Gas	6.0 l/min
Scan End	3000 m/z	Set Collision Cell RF	600.0 Vpp	Set Divert Valve	Waste

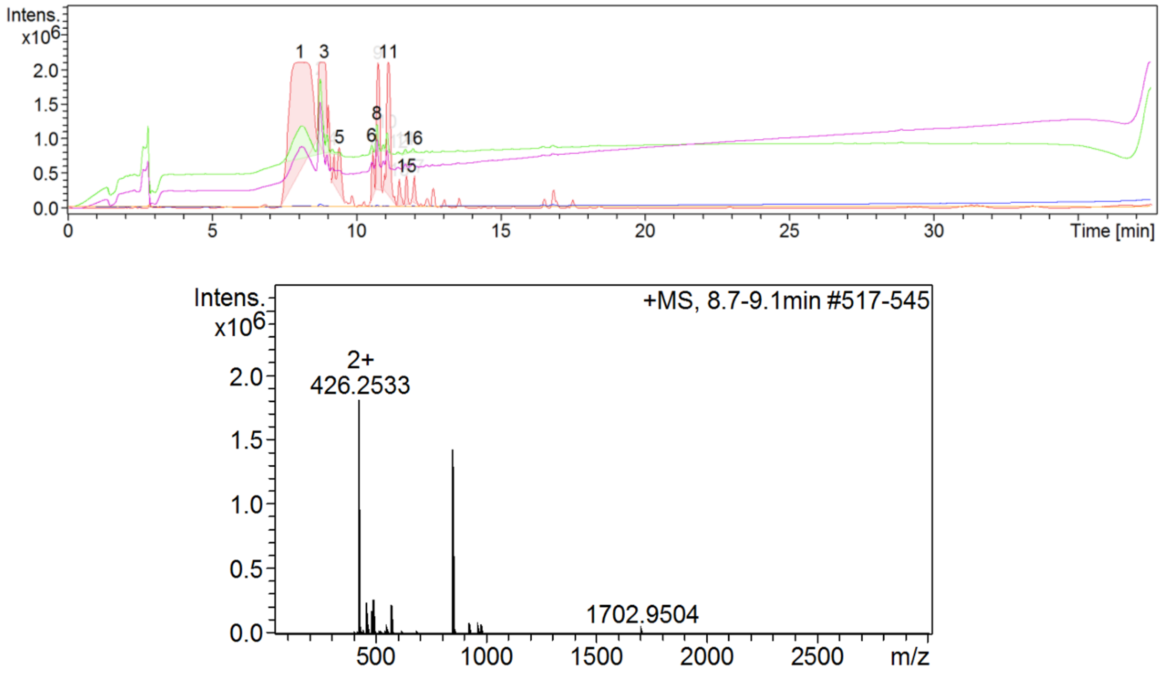

Figure S5. LC-MS spectrum of [22]YIEALDK[28] fragment.

Acquisition Parameter

Source Type	ESI	Ion Polarity	Positive	Set Nebulizer	2.4 Bar
Focus	Not active	Set Capillary	4500 V	Set Dry Heater	250 °C
Scan Begin	50 m/z	Set End Plate Offset	-500 V	Set Dry Gas	6.0 l/min
Scan End	3000 m/z	Set Collision Cell RF	600.0 Vpp	Set Divert Valve	Waste

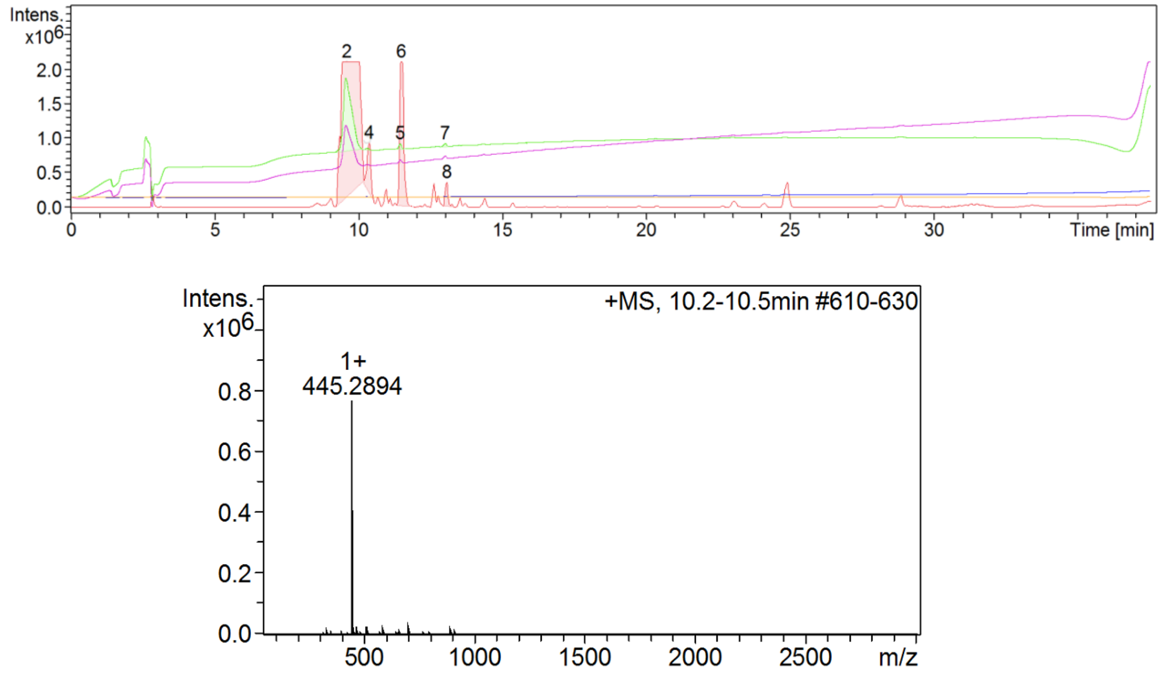

Figure S6. LC-MS spectrum of [23]IEAL[26] fragment.

Acquisition Parameter

Source Type	ESI	Ion Polarity	Positive	Set Nebulizer	2.4 Bar
Focus	Not active	Set Capillary	4500 V	Set Dry Heater	250 °C
Scan Begin	50 m/z	Set End Plate Offset	-500 V	Set Dry Gas	6.0 l/min
Scan End	3000 m/z	Set Collision Cell RF	600.0 Vpp	Set Divert Valve	Waste

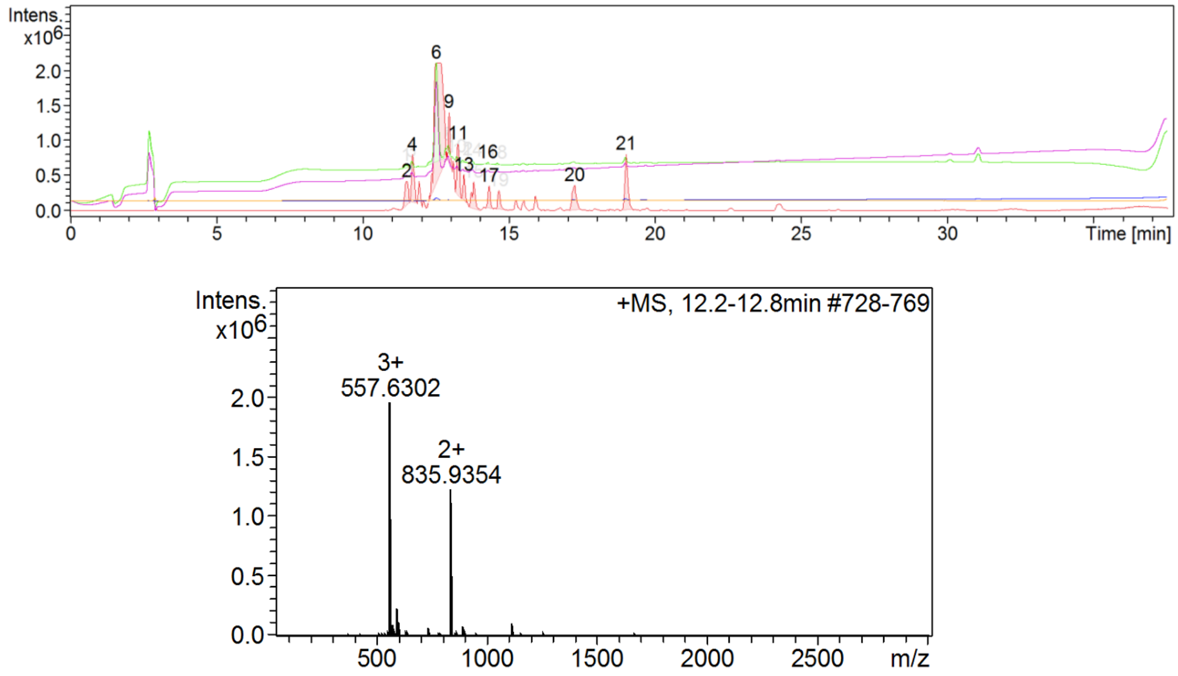

Figure S7. LC-MS spectrum of [34]VVGYIGERCQYRDL[47] fragment.

Acquisition Parameter

Source Type	ESI	Ion Polarity	Positive	Set Nebulizer	2.4 Bar
Focus	Not active	Set Capillary	4500 V	Set Dry Heater	250 °C
Scan Begin	50 m/z	Set End Plate Offset	-500 V	Set Dry Gas	6.0 l/min
Scan End	3000 m/z	Set Collision Cell RF	600.0 Vpp	Set Divert Valve	Waste

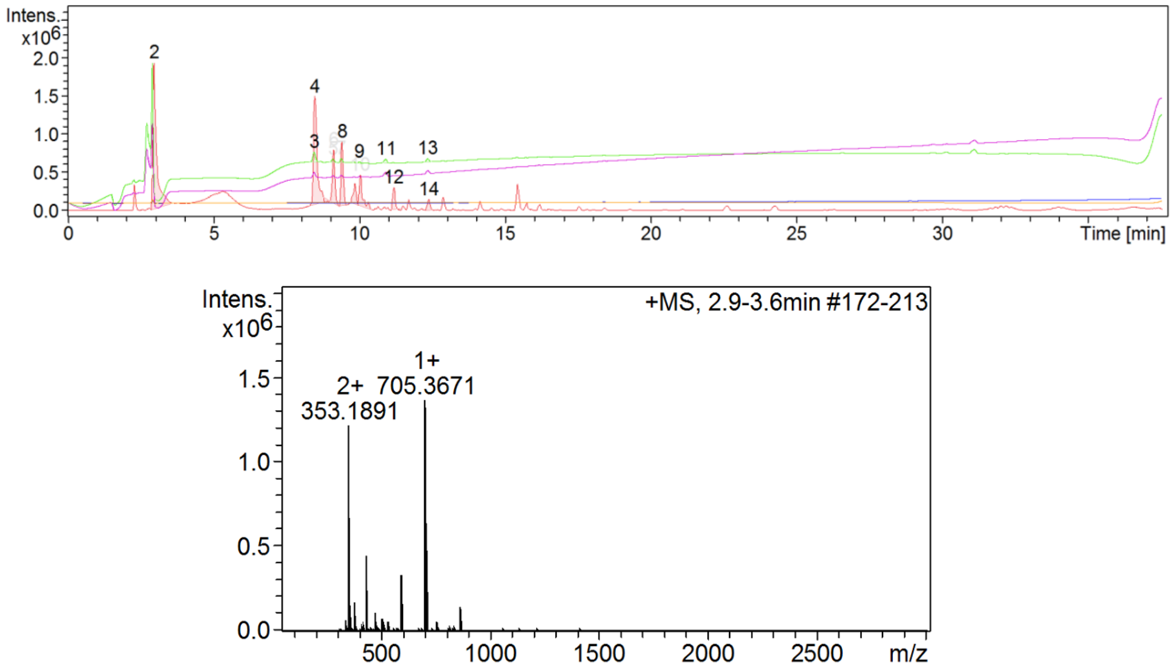

Figure S8. LC-MS spectrum of [38]IGERCQ[43] fragment.

Acquisition Parameter

Source Type	ESI	Ion Polarity	Positive	Set Nebulizer	2.4 Bar
Focus	Not active	Set Capillary	4500 V	Set Dry Heater	250 °C
Scan Begin	50 m/z	Set End Plate Offset	-500 V	Set Dry Gas	6.0 l/min
Scan End	3000 m/z	Set Collision Cell RF	600.0 Vpp	Set Divert Valve	Waste

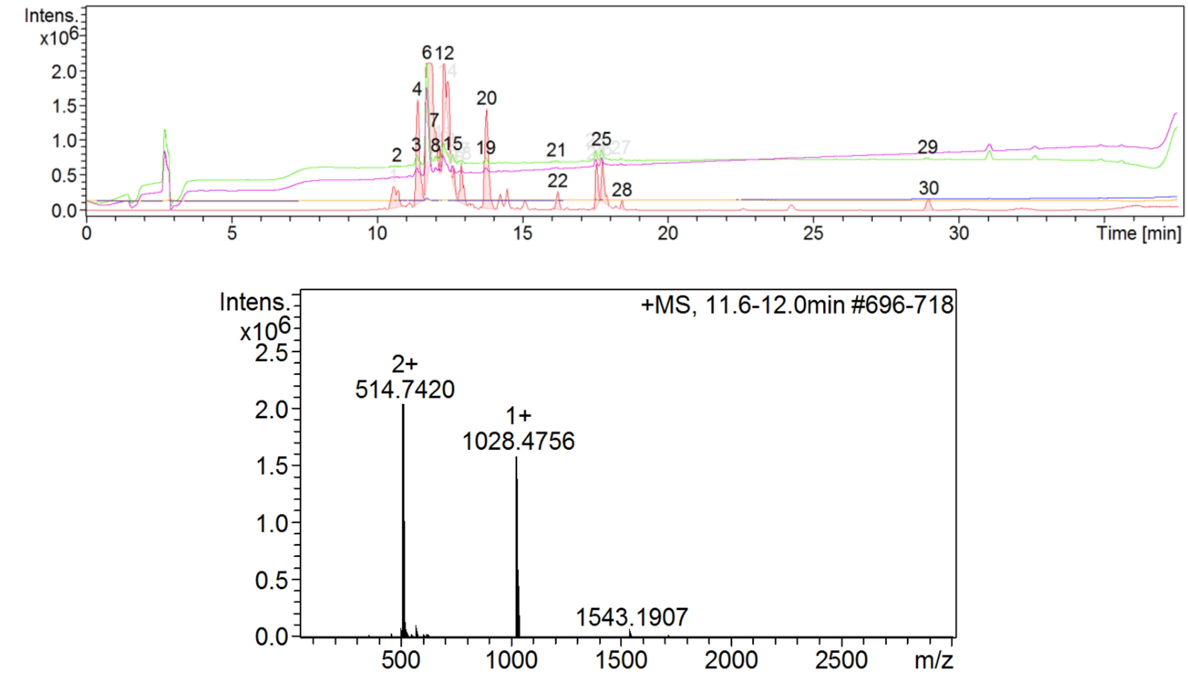

Figure S9. LC-MS spectrum of [26]LDKYACNCV[34] fragment.

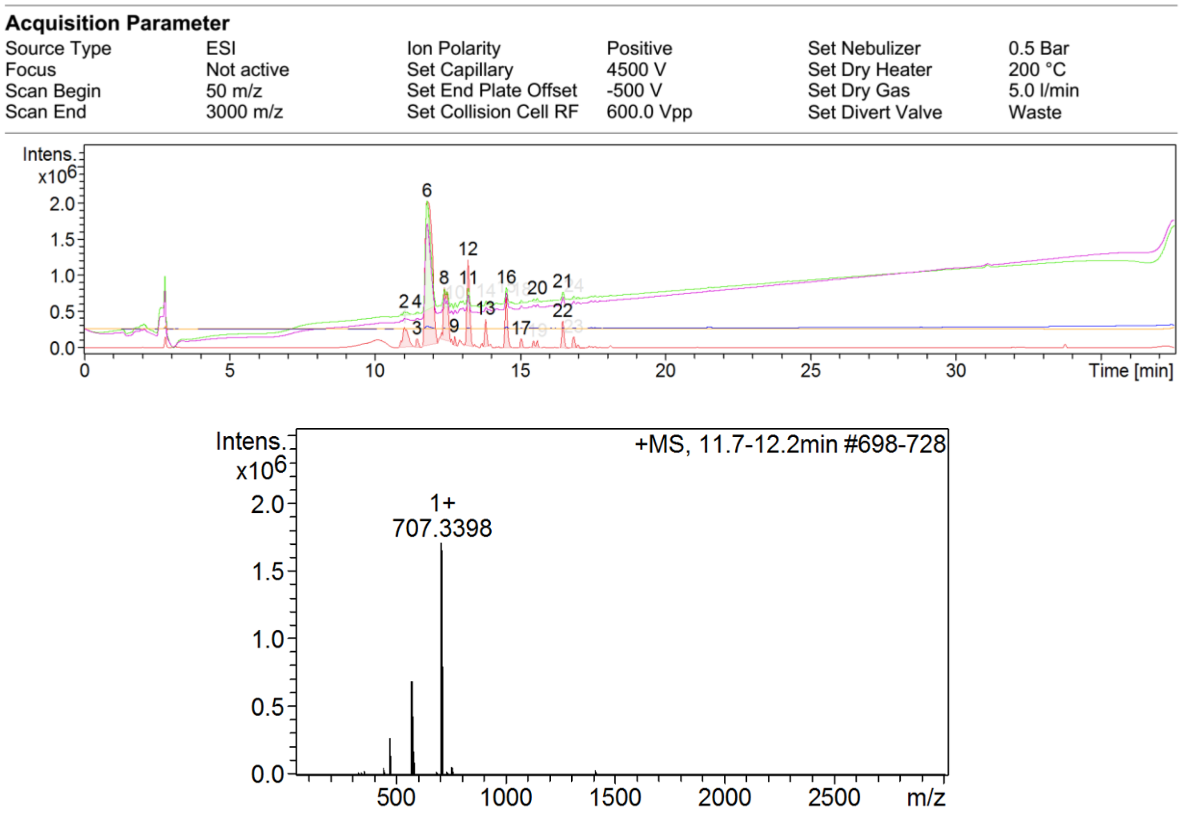

Figure S10. LC-MS spectrum of ^{10}HDGYCL^{15} fragment.

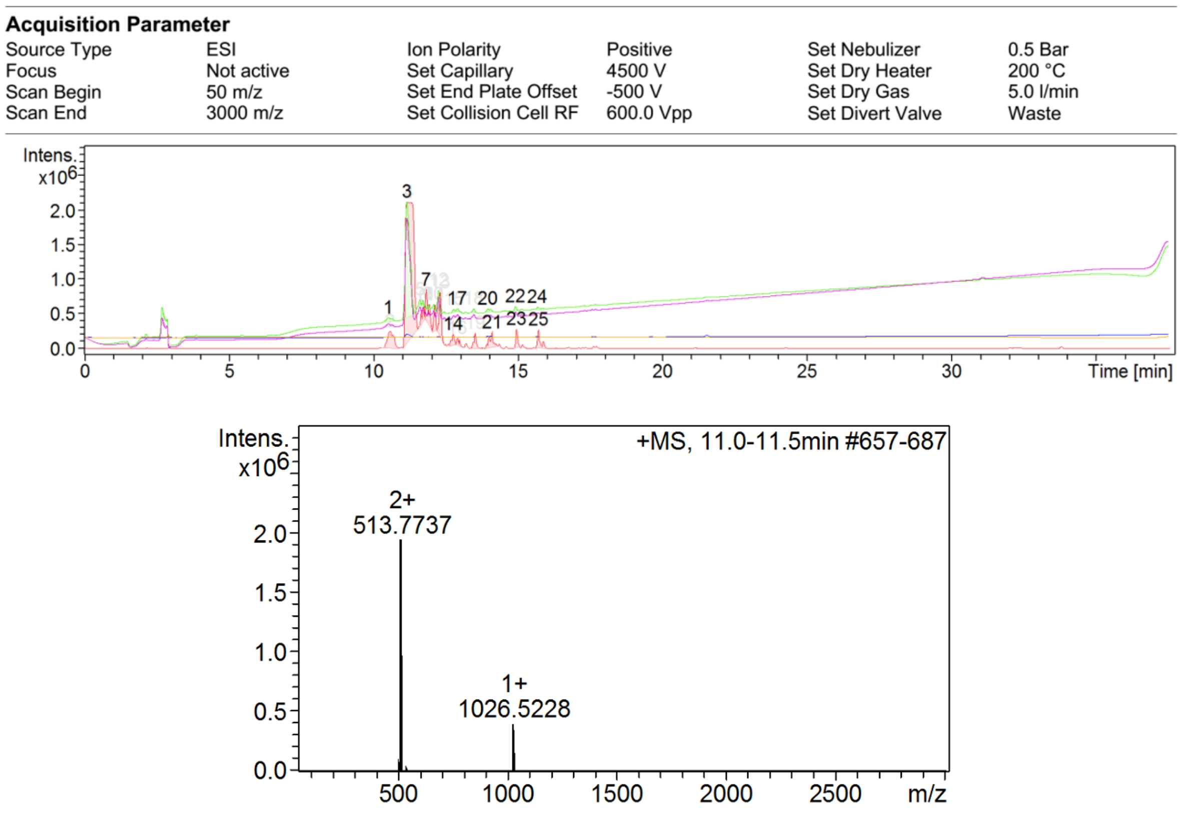

Figure S11. LC-MS spectrum of ^{10}HDGYCL^{15}+^{29}Y+^{41}R fragment.

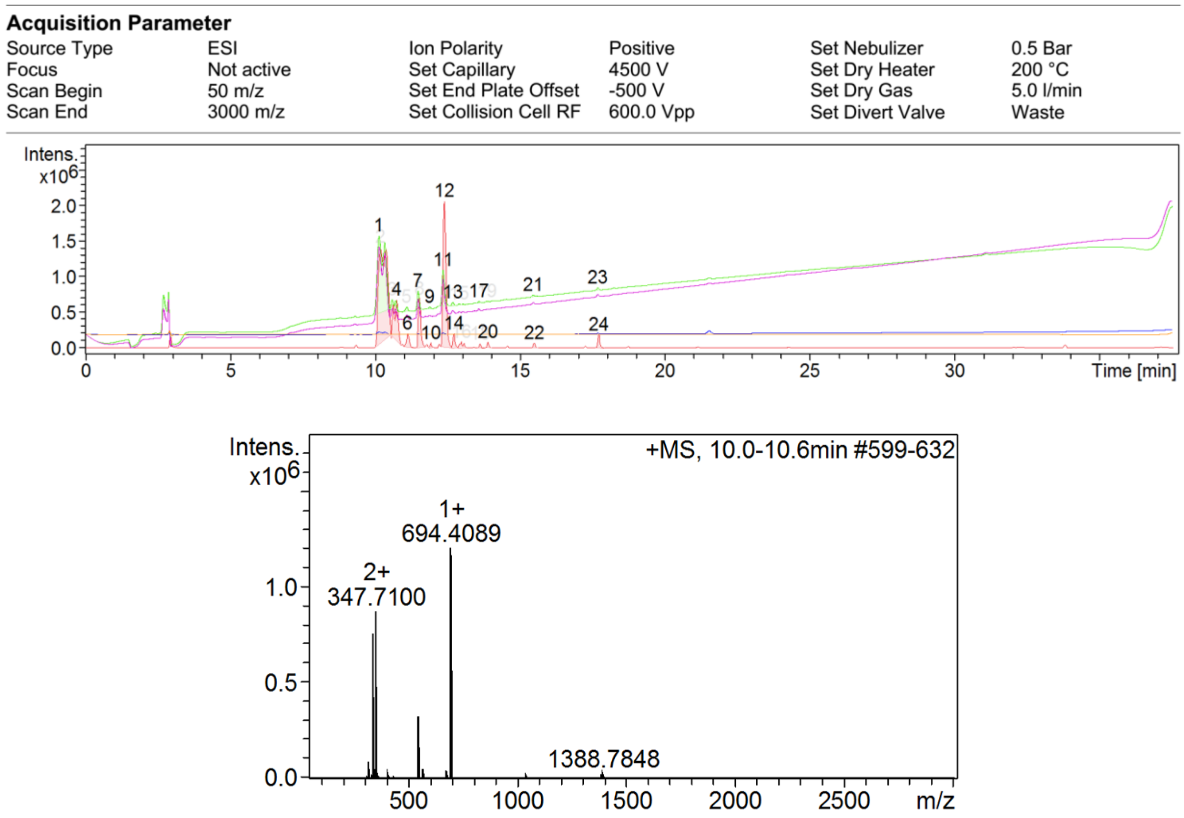

Figure S12. LC-MS spectrum of [43]QYRDL[47]fragment.

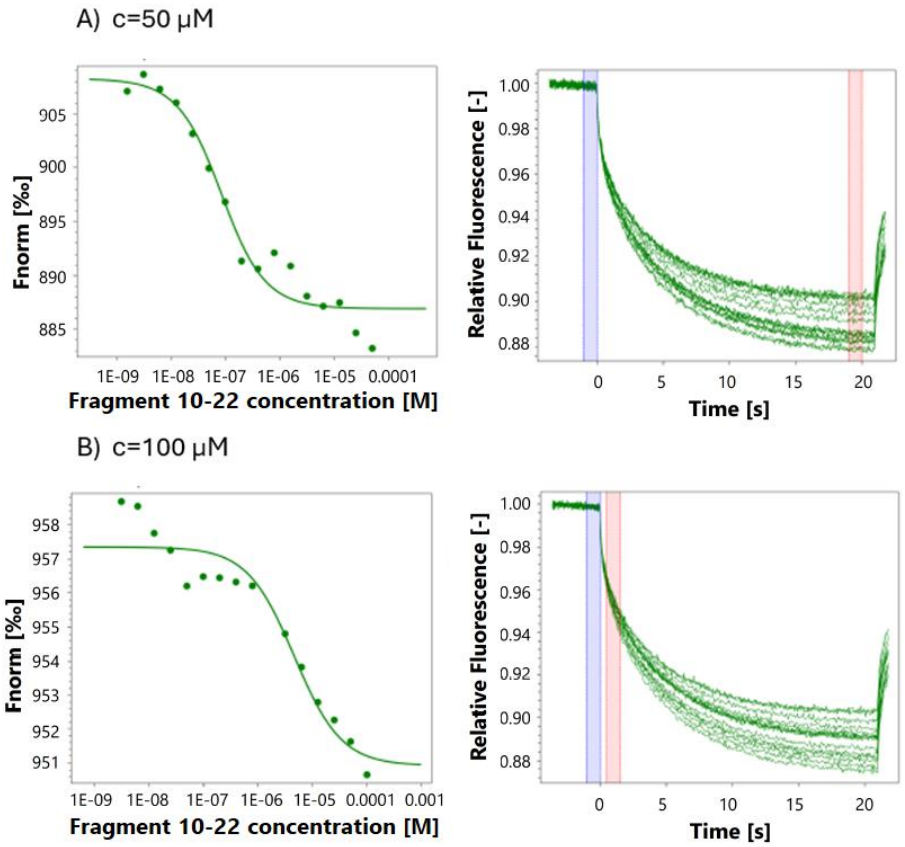

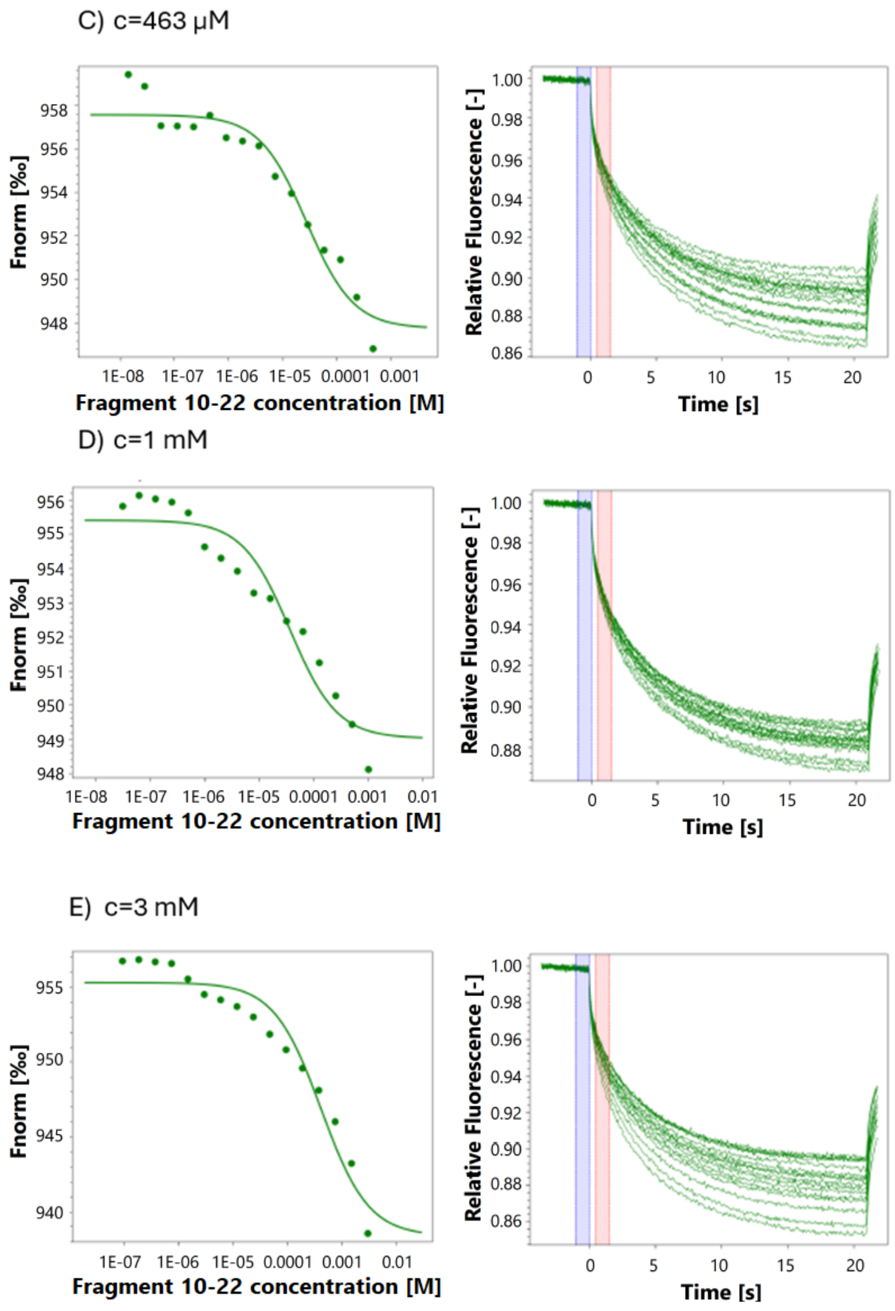

Figure S13. MST analysis for fragment 10-22 (^{10}HDGYCLHDGVCMY^{22}) at different concentrations. Assay concentrations: A) c = 50 µM, B) c = 100 µM, C) c = 463 µM, D) c = 1 mM, E) c = 3 mM.

A) c=10 µM

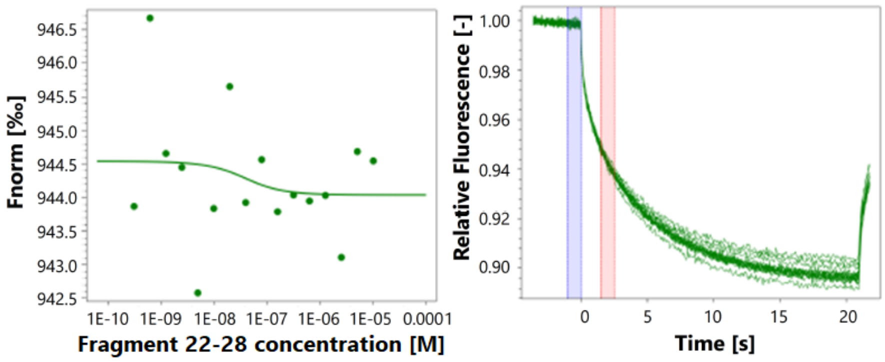

B) c=250 µM

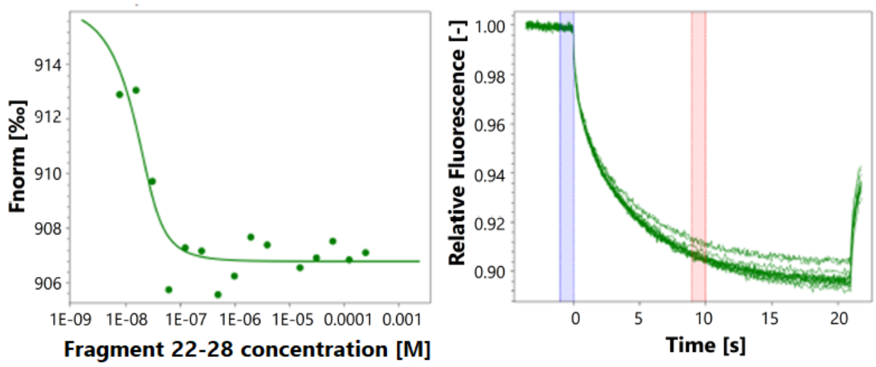

C) c=500 µM

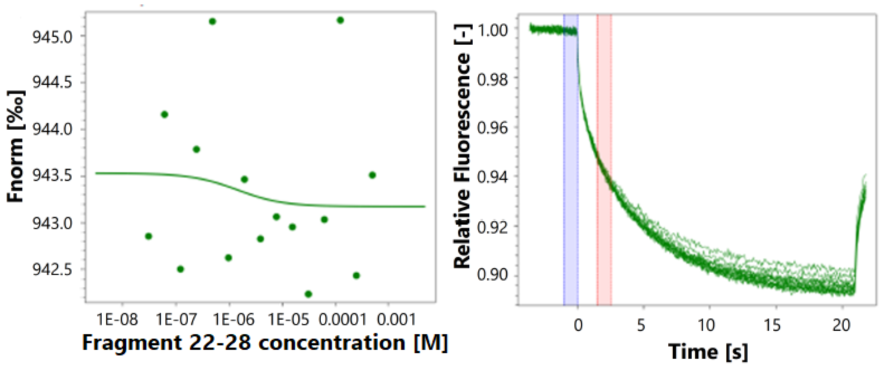

D) c=3 mM

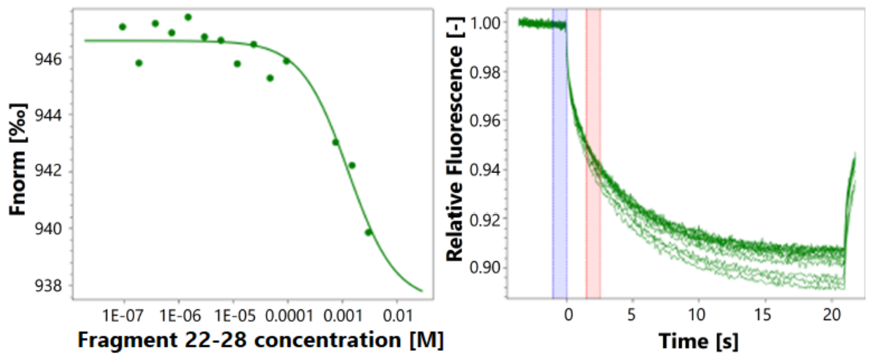

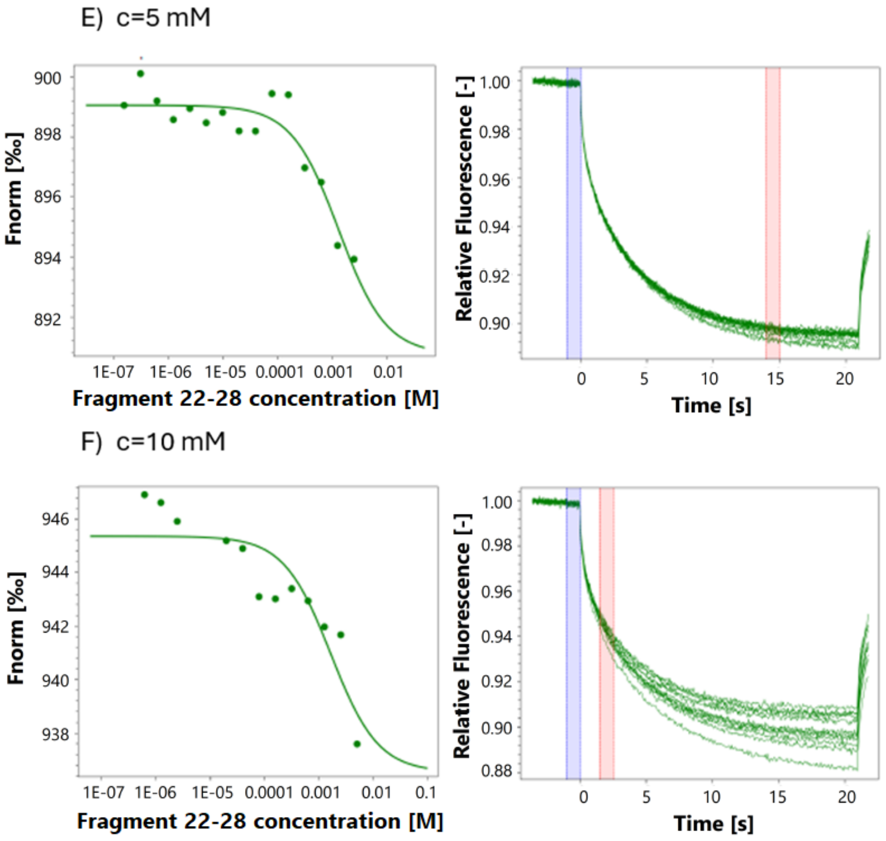

Figure S14. MST analysis for fragment 22-28 (^{22}YIEALDK^{28}) at different concentrations. Assay concentrations: A) c = 10 µM, B) c = 250 µM, C) c = 500 µM, D) c = 3 mM, E) c = 5 mM, F) c = 10 mM.

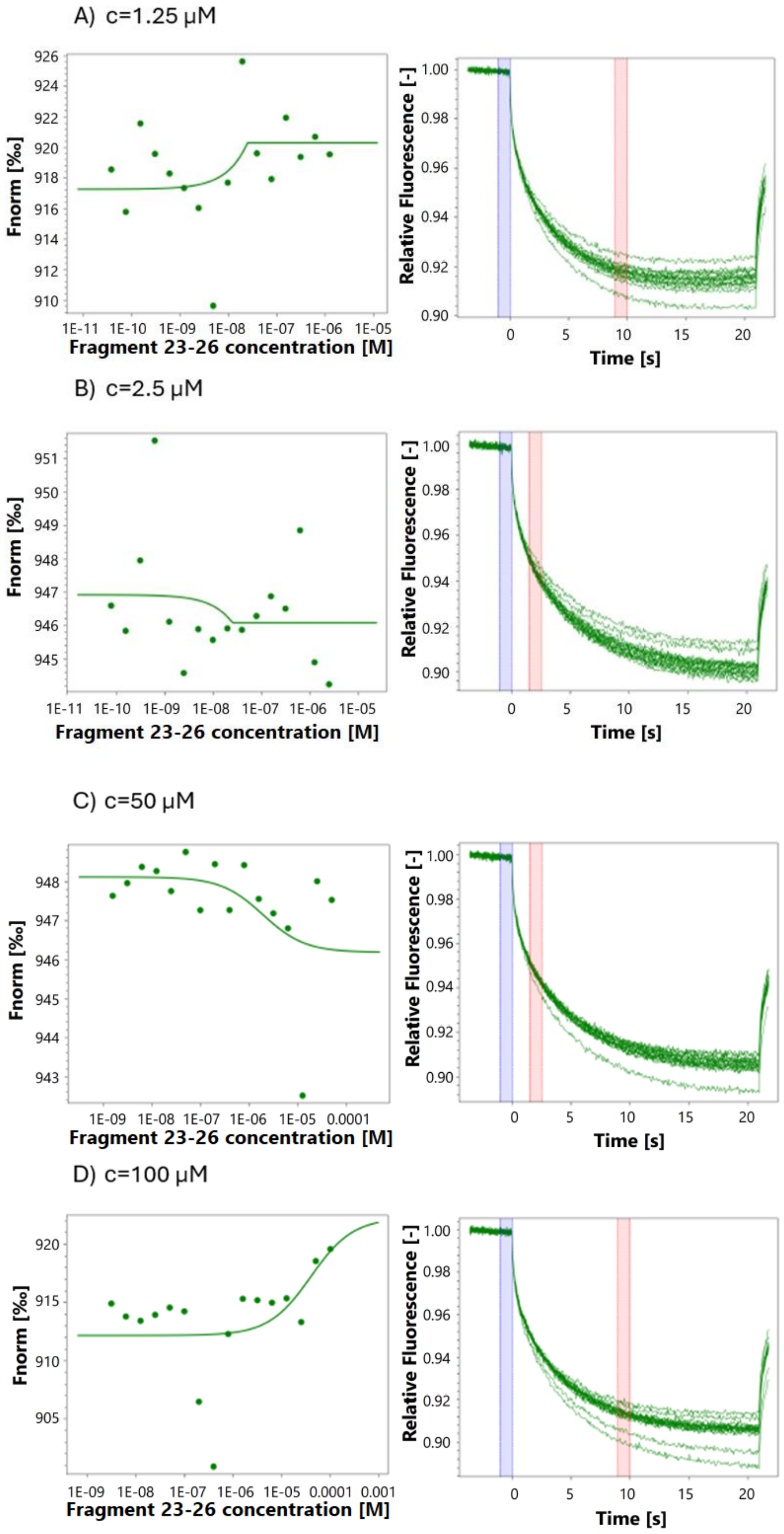
A) c=1.25 μM
Fnorm [‰]
Fragment 23-26 concentration [M]
Relative Fluorescence [-]
Time [s]
B) c=2.5 μM
Fnorm [‰]
Fragment 23-26 concentration [M]
Relative Fluorescence [-]
Time [s]
C) c=50 μM
Fnorm [‰]
Fragment 23-26 concentration [M]
Relative Fluorescence [-]
Time [s]
D) c=100 μM
Fnorm [‰]
Fragment 23-26 concentration [M]
Relative Fluorescence [-]
Time [s]

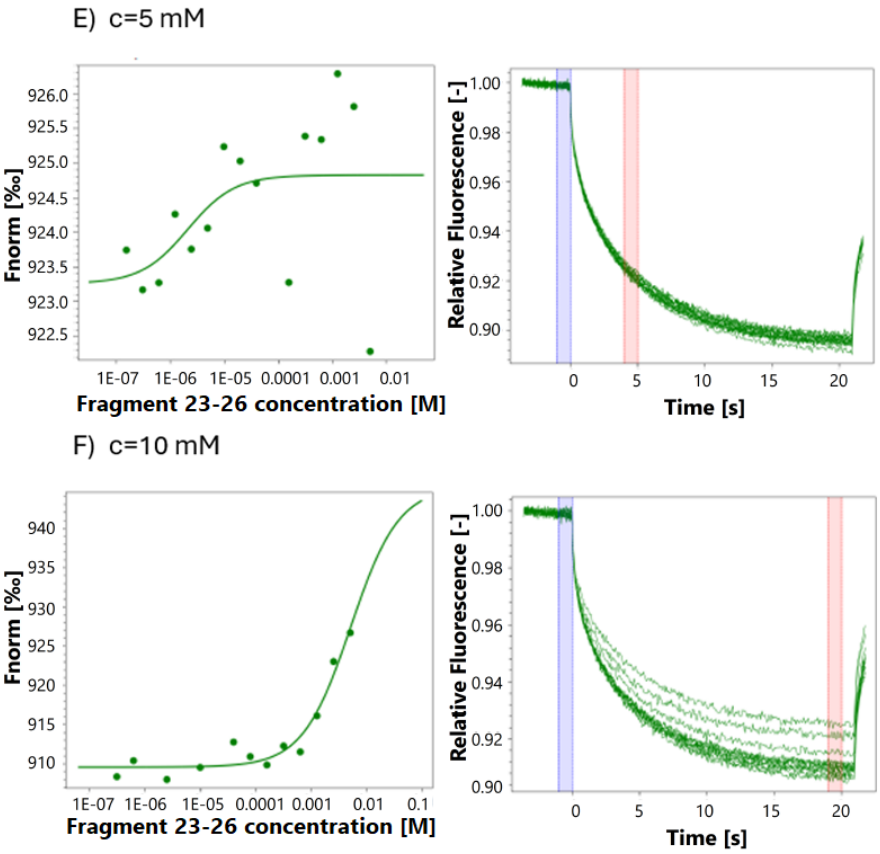

Figure S15. MST analysis for fragment 23-26 (^{23}IEAL^{26}) at different concentrations. Assay concentrations: A) c = 1.25 µM, B) c = 2.5 µM, C) c = 50 µM, D) c = 100 µM, E) c = 5 mM, F) c = 10 mM.

A) c=50 μM

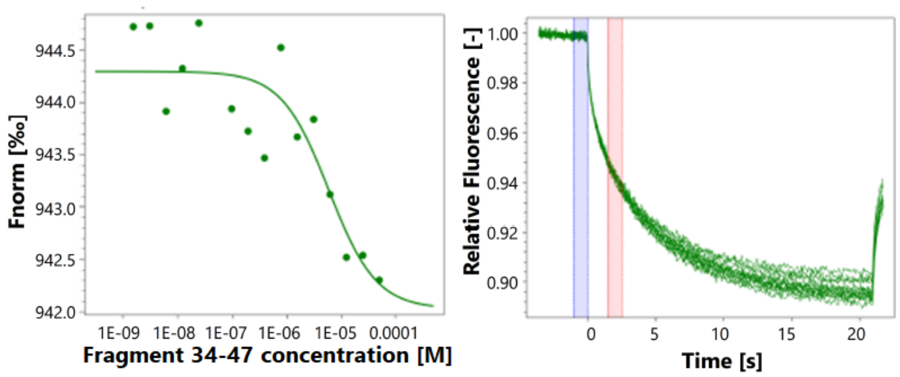

B) c=141 μM

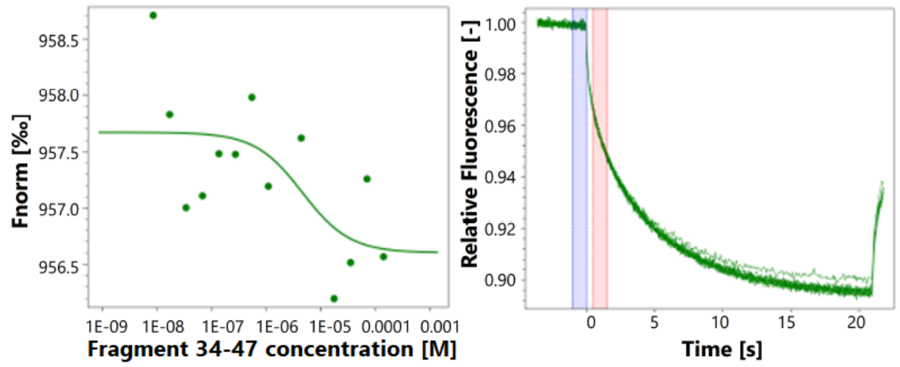

C) c=350 μM

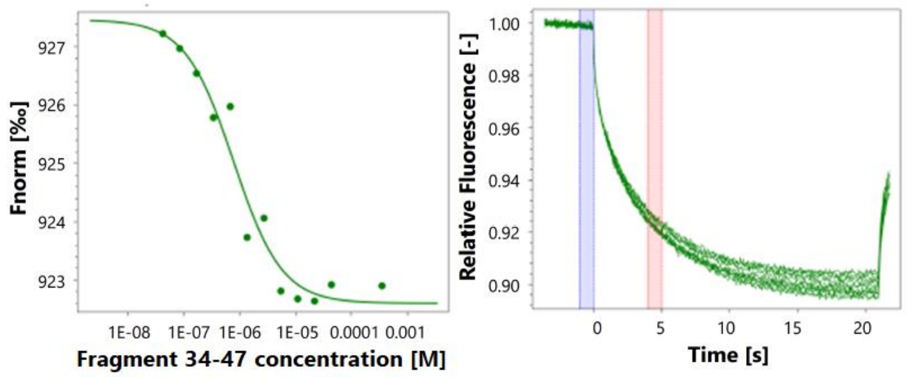

D) c=705 μM

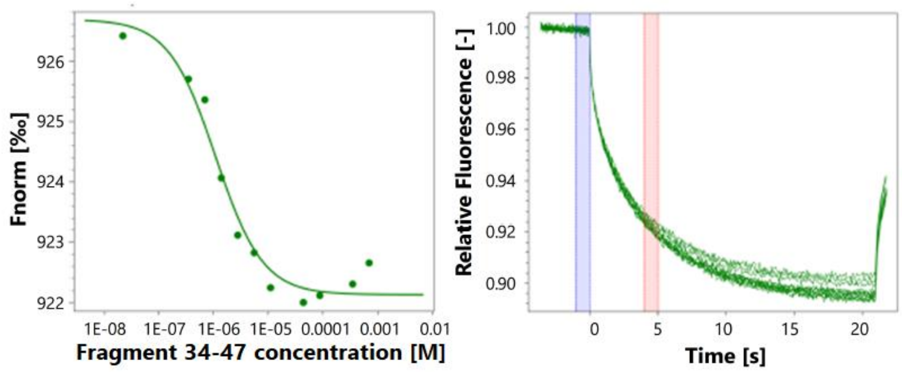

E) c=1 mM

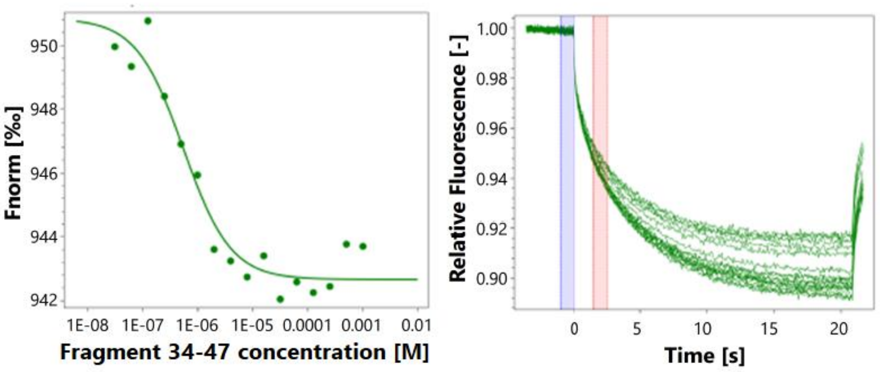

Figure S16. MST analysis for fragment 34-47 ([34]VVGYIGERCQYRDL[47]) at different concentrations. Assay concentrations: A) c = 50 µM, B) c = 141 µM, C) c = 350 µM, D) c = 705 µM, E) c = 1 mM.

A) c=10 µM

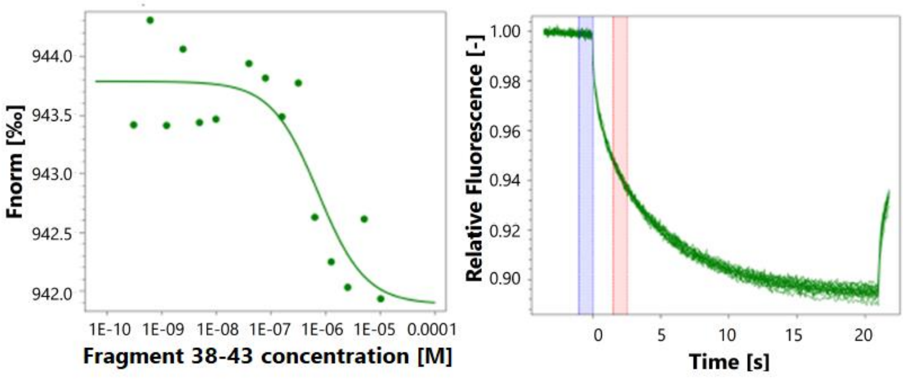

B) c=500 µM

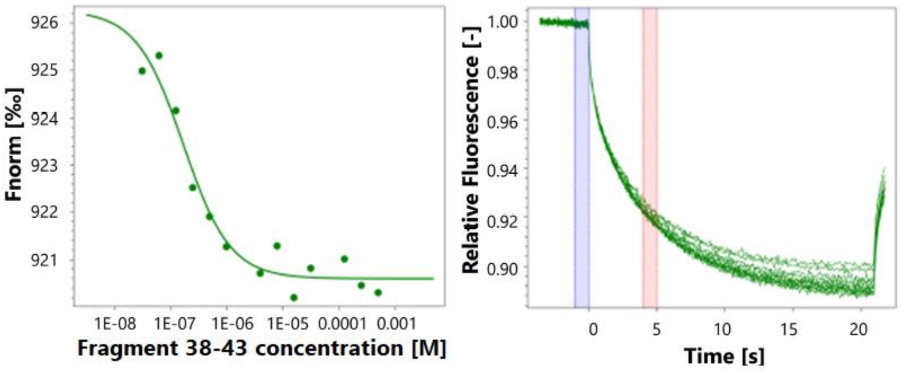

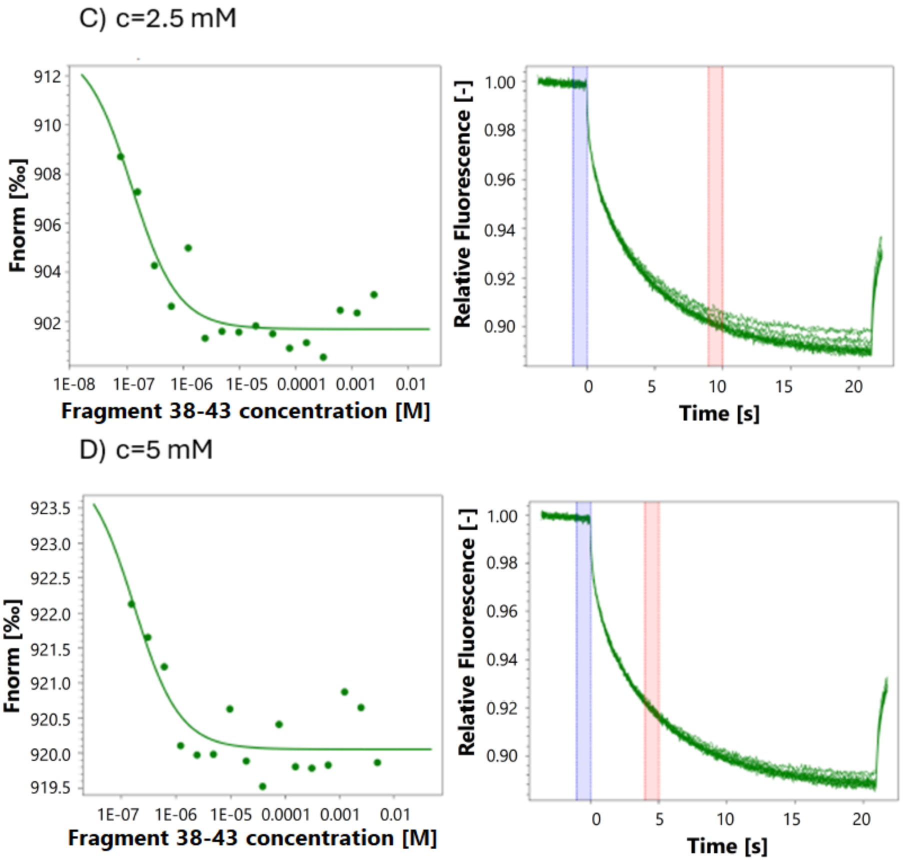

Figure S17. MST analysis for fragment 38-43 ([38]IGERCQ[43]) at different concentrations. Assay concentrations: A) c = 10 µM, B) c = 500 µM, C) c = 2.5 mM, D) c = 5 mM.

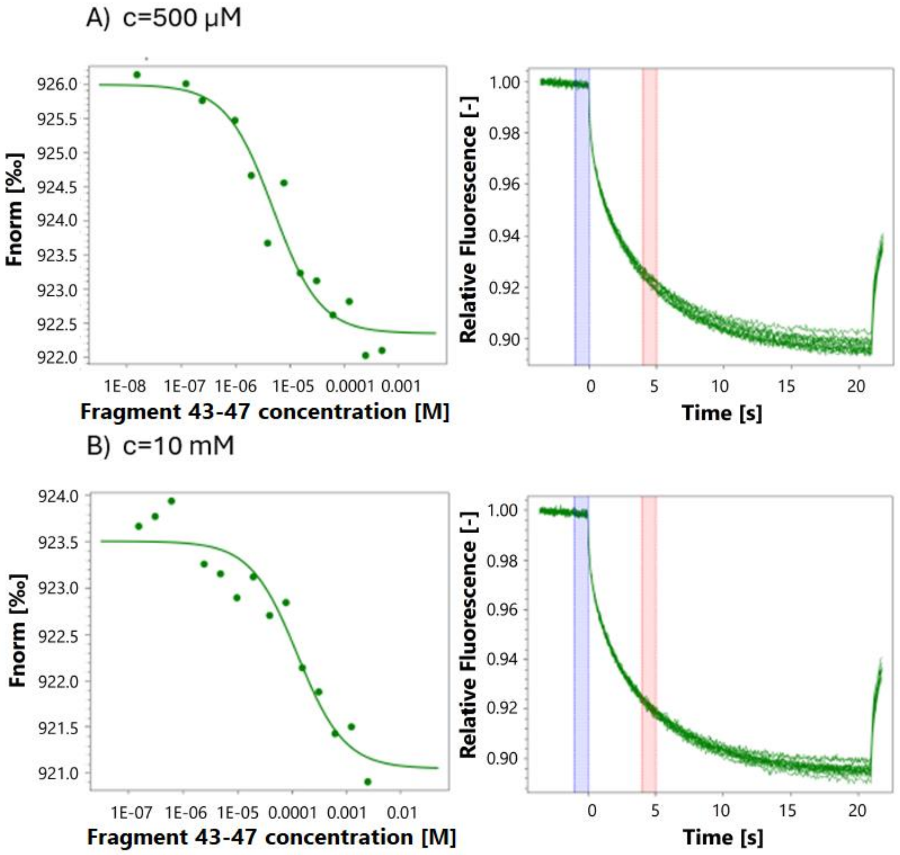

Figure S18. MST analysis for fragment 43-47 (^{43}QYRDL^{47}) at different concentrations. Assay concentrations: A) c = 500 µM, B) c = 10 mM.

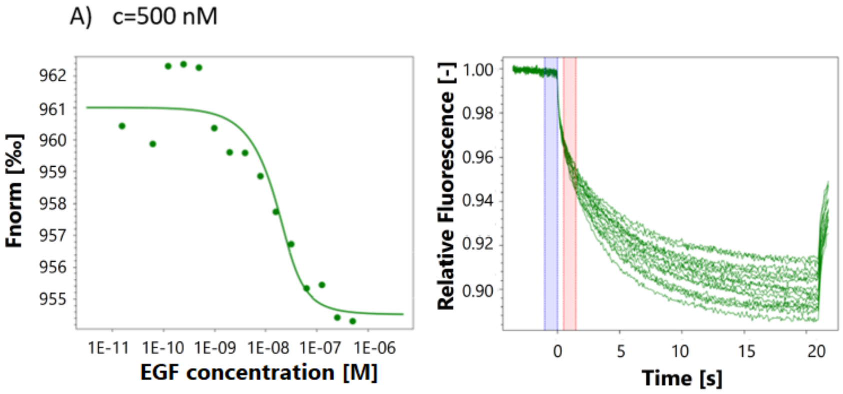

Figure S19. MST analysis for EGF. Assay concentrations: A) c = 500 nM.

---

[1] J. Fraczyk, M. Walczak, and Z. J. Kaminski, "New methodology for automated SPOT synthesis of peptides on cellulose using 1,3,5-triazine derivatives as linkers and as coupling reagents," *J. Pept. Sci.*, vol. 24, no. 2, pp. 1–12, 2018, doi: 10.1002/psc.3063.

[2] B. Kolesinska, K. K. Rozniakowski, J. Fraczyk, I. Relich, A. M. Papini, and Z. J. Kaminski, "The Effect of Counterion and Tertiary Amine on the Efficiency of *N* -Triazinylammonium Sulfonates in Solution and Solid-Phase Peptide Synthesis," *European J. Org. Chem.*, vol. 2015, no. 2, pp. 401–408, Jan. 2015, doi: 10.1002/ejoc.201402862.
